# Changes in mental health in the German child and adolescent population during the COVID-19 pandemic – Results of a rapid review

**DOI:** 10.25646/10761

**Published:** 2023-02-01

**Authors:** Robert Schlack, Laura Neuperdt, Stephan Junker, Sophie Eicher, Heike Hölling, Julia Thom, Ulrike Ravens-Sieberer, Ann-Kristin Beyer

**Affiliations:** 1 Robert Koch Institute, Berlin Department of Epidemiology and Health Monitoring; 2 University Medical Center Hamburg-Eppendorf, Clinic for Child and Adolescent Psychiatry, Psychotherapy and Psychosomatics, research devision ‘Child Public Health’

**Keywords:** COVID-19 PANDEMIC, MENTAL HEALTH, CHILDREN AND ADOLESCENTS, GERMANY, RAPID REVIEW

## Abstract

**Background:**

This rapid review examines changes in the mental health of the German child and adolescent population during the COVID-19 pandemic.

**Methods:**

The basis are 39 publications, which were identified by means of systematic literature search (until 19.11.2021) and manual search. The databases of the included publications were systematized with regard to their representativeness for the general population, and the indicators used were categorized with regard to the depicted constructs and their reliability.

**Results:**

The large majority of the studies took place at the beginning of the pandemic until the summer plateau 2020. Representative studies mainly reported high levels of pandemic-related stress, increases in mental health problems, and negative impacts on the quality of life. Non-representative studies showed mixed results. Vulnerable groups could only be identified to a limited extent. Both routine and care-related data showed declines in the outpatient and inpatient service utilisation during the various waves of the pandemic followed by catch-up effects. Children and adolescents turned out to be more vulnerable during the pandemic compared to adults, but their stress levels varied with the waves of the pandemic and the related containment measures.

**Conclusions:**

A future forward-looking crisis and pandemic management requires a close-knit and continuous surveillance of the mental health of children as well as an improved identification of risk groups.

## 1. Introduction

The beginning of the COVID-19 pandemic presented unprecedented challenges to the societies worldwide. In Germany, far-reaching non-pharmaceutical containment measures were introduced in mid-March 2020, shortly after the World Health Organisation had declared the SARS-CoV-2 epidemic to be a pandemic on March 11, 2020 [1]. These measures included social distancing, cancellation of large-scale events, travel warnings, quarantine, recommendations or obligations to work at the home, as well as temporary closures of playgrounds, day-care centres, and schools. Due to their specific developmental vulnerability, children and adolescents were particularly affected by many of these measures, which initially served in particular to protect older or chronically ill individuals [2, 3]. For instance, the closures of day-care centres and of schools resulted in educational restrictions, loss of daily routine, or lack of or limited physical activity. Leisure activities, social and family contacts to grandparents, relatives, and friends were likewise restricted. Forced proximity due to the containment measures and/or pandemic-related economic difficulties in the families increased the risks for familial tensions and disputes and presumably also for domestic violence and child abuse and neglect [4]. Children and adolescents were also reported of being afraid of an own COVID-19 infection or the infection of a close family member (parents, grandparents) or their illness or death [5, 6].

Putative impacts of the pandemic as well as impacts of non-pharmacological containment measures on the population mental health had been addressed in the international literature right from the start, more specifically, however, for adults than for children and adolescents [7–10]. In December 2020, Schlack et al. [4] presented a non-systematic, narrative literature review on the mental health of children and adolescents during the pandemic. It combined respective data and empirical results from the first pandemic wave for Germany until July 2020. At that point, however, these results were often only available as advance notifications or as press reports or press releases. These first findings suggested a high level of pandemic-related mental stress, fears and worries as well as a decline in the quality of life among children and adolescents and their families. Children and adolescents from socioeconomically disadvantaged families, from families with migration background, as well as children with pre-existing mental disorders appeared particularly affected [4]. After almost two years and three additional waves of the pandemic, a new review of the literature on the population-based mental health of children and adolescents in Germany during the pandemic seemed appropriate. Thus, this paper aims at a systematic synthesis of evidence according to the methodology of a rapid review. As in a systematic review, in a rapid review the evidence is systematically synthesised, however more quickly, using methodological ‘shortcuts’ [11, 12].

In an abruptly occurring major health crisis, such as the current pandemic, authorities need quick and reliable data both about vulnerable population groups and the level of care they need [12] in order to be able to initiate measures effectively and quickly. Thereby, it is important that the data be generalisable to the general population [12, 13]. Ad-hoc conducted online surveys using self-recruiting convenience samples via the internet, social media, or mouth-to-mouth propaganda can be used best to generate information quickly and cost-effectively. However, such studies are rarely population-representative and thus subject to the risk of bias with regard to the assessment of prevalences and/or time trends. In the case of surveys focussing on psychosocial stress, for example, individuals who acutely feel stressed may be particularly motivated to participate and may thus be overrepresented in the sample [14]. Vice versa, individuals with manifest mental disorders, older people, as well as individuals with low education tend to participate less likely in online surveys [12]. In studies using convenience samples, systematic non-participation of specific population subgroups cannot be compensated by means of weighting.

The present rapid review has thus four main goals: First, to synthesise the evidence of the quantitative research relating to the mental health of children and adolescents in Germany as well as of the data of their care with regard to mental health problems and disorders during the pandemic. The regional scope of the data acquisitions was thus limited to Germany. Studies from other countries as well as studies using mixed samples with participants from several countries were excluded. German (sub-)samples, however, were considered when the results were reported separately. For not to exclude relevant publications with participants that had reached the age of majority in individual cases, studies with participants up to an upper age limit of 20 were included in the review. When studies including a broad age spectrum up to adulthood reported results for youths up to the age of 17 separately, these were likewise included. Secondly, it is to be determined how reliably and how comprehensively the mental health of children and adolescents is represented in the included publications. To do so, the indicators, tools, and inventories used in the studies were each assigned to psychological constructs and summarised in tables. Thirdly, the databases of the included studies are assessed with respect to representativeness for the general population. In order to do so, the data representing the basis for the included publications were systematised with regard to their suitability for representative statements for the respective reference population and were depicted in tables, too. Fourthly, it will be analysed, how (well) the development of the mental health of children during the pandemic is represented in German research and the German data landscape. Thus, the number and the survey periods of the data (primary and routine data) forming the basis for the included publications were correlated to key figures of the pandemic.

In the past year, a rapid review relating to the mental health of the adult population during the COVID-19 pandemic from the Unit of Mental Health at the Robert Koch-Institute had already been presented as part of the emerging mental health surveillance [16]. So as to report most uniformly and comparably across the entire age spectrum, this rapid review refers to the rapid review on the German adult population of Mauz et al. in terms of the procedure and the presentation of the results in figures and tables [15].

## 2. Methodology

The methodology of this review is based on the standardised approach for the rapid review process proposed by the German Public Health Research Network on COVID-19 (https://www.public-health-covid19.de/en/) in the context of the current pandemic [11] with reference to Tricco et al. [16]. This will be described in detail below.

### 2.1 Literature search

The literature search was performed on the basis of the PECO criteria using the following specifications: Population: Children and adolescents in Germany; Exposition: COVID-19 pandemic; Comparison: before/after or after COVID outbreak in Germany; Outcome: Mental health. The inclusion and exclusion criteria were:

#### Inclusion criteria

1. Target population: General population in Germany (as well as subgroups according to region and age)
2. Age group: Children and adolescents up to the age of 20
3. Observational period: During the COVID-19 pandemic
4. Constructs reported: Mental health as main outcome
5. Publication of a temporal comparison (compared to measurement points before the start of the pandemic or during the pandemic)
6. Publication language: German/English

#### Exclusion criteria

1. Publication type: Reviews, review articles, statements, comments, letters to the editor
2. Methodology: Purely qualitative data
3. Presentation of the study methodology: Methodology not presented with sufficient transparency
4. Evaluation design: Exclusively correlation analyses without reporting of frequencies or their changes in the general population, respectively
5. Target population: Subgroups beyond the above-mentioned sociodemographic characteristics (e.g. individuals with pre-existing specific mental disorders, excluding attention deficit/hyperactivity disorders (ADHS) and eating disorders)

#### Systematic search

The search was based on the literature database created from the library of the Robert Koch Institute in the course of the COVID-19 pandemic (As at 19.11.2021). Since the beginning of the pandemic, all publications identified by means of several search strings (Annex Table 1) in the PubMed and Embase databases as well as the additionally searched preprint servers ArRvix, BioRvix, ChemRvix, MedRvix, Preprints.org, ResearchSquare, and SSRN, are entered into this database on a weekly basis.

The literature database was searched for texts on mental health, school, and school closures as well as on domestic violence using several search strings (Annex Table 1) defined by filter terms. All texts extracted in this way were initially filtered by ‘children and adolescents’ using a fourth search string, and by their reference to ‘Germany’ using a fifth search string.

#### Manual search

Beyond the systematic search in the mentioned international databases, publications were searched in additional dissemination formats, such as reports, websites of studies, press releases, or reports from health insurance funds, as it can be assumed in view of the high need for information and the resulting rapid succession of scientific publications on COVID-19 that not all findings were already listed in there. In particular, health insurance fund data is also not necessarily published in a scientific journal. The literature search was thus extended to include the following areas:

1. Systematic search in the World Health Organization (WHO) database ‘COVID-19. Global literature on coronavirus disease’ (last update 06.12.2021; search string see Annex Table 1).
2. Websites of COVID-19 related studies in the general population, listed on the website of the German Data Forum (RatSWD) (As at 24.11.2021).
3. Press releases and current reports or studies, respectively, e.g. from service carriers and providers of the health care system (e.g. health insurance funds, outpatient care data from the National Association of Statutory Health Insurance Physicians, hospital statistics) as well as care-related primary data (last update 06.04.2022).
4. Screening publications and literature lists from the COVID-19 Public Health Research Network (last update 22.11.2021).
5. Search for relevant COVID-19 related studies and publications via search engine Google (As at 16.11.2021; search string see Annex Table 1).
6. Screening for relevant COVID-19 related studies and publications, listed on the website of the German Association for Psychiatry, Psychotherapy, Neurology, and Psychosomatics (DGPPN) (As at 15.11.2021).
7. Screening lists of references of COVID-19 related reviews, comments, and policy briefs relating to relevant studies and publications (last update 10.02.2022).

### 2.2 Title and abstract screening, full text analysis

After the systematic search, the publications extracted thereby were subjected to a title and abstract screening. Twenty percent of the extracted publications were additionally checked by two experienced individuals (RS and AKB). This did not result in any diverging assessments. In a next step, the remaining publications were subjected to a full text analysis and were classified according to their suitability for the inclusion in the review. The publications that were found during this manual search were handled analogously. All publications were checked by at least two individuals. Publications, for which an unambiguous decision could not be made initially, were discussed in the team with at least three individuals and were included or excluded in an iterative process according to the above-mentioned criteria.

### 2.3 Systematic extraction of the relevant data

The relevant data of the included publications was extracted systematically according to the above-presented criteria and was prepared in tabular form (Annex Table 3), the used indicators were identified, classified, and likewise prepared in tabular form (Annex Table 4). This was approached on the basis of the procedure Mauz et al. [15]. The data extraction was quality-assured in each case by at least two additional individuals.

### 2.4 Classification of the included publications with regard to content

Primary data acquisitions with direct information relating to possible changes in the mental health of the child and adolescent population in Germany during the COVID-19 pandemic were combined under the category I ‘Primary data on the mental health of children and adolescents in Germany in the context of the pandemic’ (see Annex Tables 3 and Annex Tables 4). Aspects of the care of children and adolescents in the context of mental health problems and disorders from published billing data from the National Association of Statutory Health Insurance Physicians, statutory health insurance funds, and the Federal Office of Statistics as well as from primary data acquisitions with care reference were combined under the category II ‘Routine data and care-related primary data’.

### 2.5 Classification of the indicators mental health

The indicators, tools, and inventories relating to the survey on the mental health of children and adolescents, which were reported in the included publications, were classified, analogously to the approach in Mauz et al. [15], but adapted to child and adolescent age, initially according to their higher-level outcome areas as follows (see also Annex Table 4):

Outcome type (a): Indicators of positive mental health,
Outcome type (b): Indicators of mental distress,
Outcome type (c): Indicators of acute symptoms of a mental disorder,
Outcome type (d): Indicators relating to the experience of violence
Outcome type (e): Indicators from routine data, and
Outcome type (f): Indicators from care-related primary data acquisitions.

Another contextual breakdown according to the respective identified constructs as well as the used inventories and survey tools (outcome type a–d) or the respective identified care areas (outcome type e and f), was made within the classification of the outcome types. On the one hand, these assignments are to make it possible to assess, how comprehensively the mental health among children and adolescents in Germany during the COVID-19 pandemic was captured, and, on the other hand (with regard to the use of standardised and validated tools), the reliableness, with which the contextual outcome areas were surveyed.

### 2.6 Systematisation according to the type of the data basis (study types)

Insofar as the data bases of the included publications were primary data acquisitions, a systematisation according to the acquisition design of the respective databases into the study types A–G was made based on the classification of Mauz et al. [15], but with subject-related adaptations and extensions (Annex Table 2).

Publications were assigned to the study type A, when its data basis allowed comparisons with prevalence or traits either from the pre-pandemic time period or at different points in time during the pandemic on the basis of a repetitive comprehensive survey or a repetitive cross-sectional sample drawn by means of a random process, or a population-representative quota sampling.

Publications, the data basis of which represented a representative trend study, based on a repetitive sample drawn by means of a random process or by means of a population-representative quota process from an access panel, were assigned to study type B.

Publications, the data basis of which was based on a one-time comprehensive survey or cross-sectional study, based on a sample drawn by means of a random process from a reference population, an access panel, or a population-representative quota sampling, were assigned to study type C, in turn.

Lastly, publications, the data basis of which was a longitudinal study with a representative initial sample at the first survey point, which allows drawing conclusions to population-based intra-individual changes, were assigned to study type D. According to the procedure of Mauz et al. [15], the study types A–D were classified as being methodologically more reliable with regard to their informative value for the general population. In contrast, cross-sectional studies with non-representative, self-selected samples (i.e., convenience samples; study type E) were classified as being methodologically reliable only to a limited extent with regard to their population-representative statements, as well as repeated cross-sectional surveys (study type F) or longitudinal studies (study type G) on the basis of non-representative initial samples.

Routine data was assigned to the data sources, which were deemed methodologically more reliable with regard to their statements about the general population, with the informative value being comparable with study type A–D. They result as part of documentation and billing processes of statutory health insurance funds or in official statistics, representing comprehensive surveys of the respective population, and are thus representative for it. In the case of statutory health insurance funds, they are representative, for instance, for the respective insured clientele. In the case of publications from the data of individual health insurance funds, a weighting (e.g. by age and sex) is also often made using the German microcensus data in order to obtain an adaptation to the general population [see 17]. Publications with results from care-related primary data acquisitions were systematised in the same way as the primary data acquisition of category I. Even if they do not provide any primary data in the strict sense, data from networks of medical practices [18, 19] were also included in the care-related primary data acquisition in this review because – contrary to, for example, the routine data from statutory health insurance funds – they represent a selective and not a comprehensive survey of the respective population.

### 2.7 Course of the pandemic and temporal correspondence of the data acquisitions

To analyse to what extent the data acquisitions, on which the included publications are based, reflect the course of the pandemic since March 2020, and for which phases of the pandemic they allow conclusions to be drawn, the observational periods of the respective data acquisitions for all included publications were determined and were compared with the development of the incidences and death rates during the COVID-19 pandemic (Figure 2). Based on the continuously updated division of the pandemic phases according to Schilling et al. [20, 21] and Tolksdorf et al. [22], the course of the pandemic until the end of the inclusion period of the review on 19.11.2021 was divided in retrospect into six phases:

1. Wave 1 from mid-March to mid-May,
2. Summer plateau 2020 from mid-May to the end of September 2020,
3. Wave 2 from the beginning of October 2020 to the end of February 2021,
4. Wave 3 from the beginning of March to mid-June 2021,
5. Summer plateau 2021 from mid-June to mid-July 2021, as well as
6. Wave 4 starting in August 2021.

As with Mauz et al. [15], the observational periods of the data acquisitions, on which the included publications are based, were specified in intervals of half months over the total period of the pandemic. Periods, for which the data for at least seven days were available in the respective half of the month, are presented. If this did not apply for any half of the month, the study was assigned to the period with the most study days.

## 3. Results

### 3.1 Literature search

**Figure 1:**
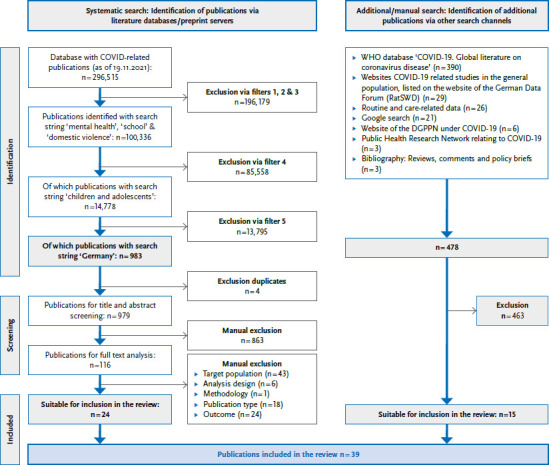
Flowchart on the inclusions and exclusions of the literature search Source: own table

At the end of the multi-stage inclusion and exclusion process according to the above-mentioned criteria, 983 publications relating to Germany were extracted according to these criteria from the systematic search (as of: 19.11.2021), 24 of which were suitable for the inclusion in the review after manual exclusion. A total of 478 publications (As at 10.02.2022) were found using the manual search. Fifteen of them were suitable for an inclusion in the review. A total of 39 publications could thus be included in the review (Figure 1).

### 3.2 Classification of the included publications according to context and systematisation according to study types

A total of 28 publications from 22 primary data acquisitions (category I ‘Primary data on the mental health of children and adolescents in Germany in the context of the pandemic’), five publications based on four routine data sources, and seven publications from six care-related primary data acquisitions (category II ‘Routine data and care-related primary data’) could be included in the review.

With regard to category I ‘Primary data on the mental health of children and adolescents in Germany in the context of the pandemic’, a total of two publications from two trend studies with random samples from the general population (study type A), nine publications from three trend studies with random sample from an access panel (study type B), and one publication each from five representative cross-sectional studies (study type C) with six data bodies (one study reported from two different data bodies, medical clinic and school sample [23]) were included in the review. Three studies with one publication each originated from population-based longitudinal studies (study type D). Five cross-sectional studies with one publication each (study type E), a repetitive cross-sectional study with one publication (study type F), and three longitudinal studies with one publication each (study type G) were based on non-representative non-probability samples. A total of 19 publications from 13 data bodies are thus available with regard to category I as data bodies (study types A, B, C, and D), which are assessed as being comparatively reliable with respect to their informative value about the general population. Nine publications from nine data bodies belonged to the study types E, F, and G, which are rather more susceptible to bias with regard to their suitability for representative statements for the general population.

From the routine data, five publications from four data sources are available in category II ‘Routine data and care-related primary data’. A total of four publications from three representative cross-sectional studies (study type C) and three repetitive cross-sectional studies with non-probability samples with one publication each (study type F) were included in the case of the care-related primary data. Four publications from three studies, which are assessed to be reliable with respect to their informative value about the general population, were available with regard to the care-related data. Three publications from three data bodies are considered here to be the study types, which are rather more susceptible to bias in this respect.

In consideration of all included publications, more than two thirds (28 of 39) of the publications or two thirds of the underlying data sources (20 von 32), respectively, can thus be assigned to the study types A, B, C, and und D, which are assessed to be rather reliable with regard to their informative value about the general population, or to the routine data, respectively.

### 3.3 Result synthesis according to indicator type and data basis

#### (a) Indicators of positive mental health

A total of 16 of the 39 publications included in the review contained indicators of the positive mental health from a total of twelve data bodies. These could be assigned to three higher-level constructs: quality of life/wellbeing, life satisfaction, and general health (Annex Tables 3 and Annex Tables 4). Results from publications of the study types A–D as well as E and G are available for indicators of positive mental health. In the respective studies, mainly standardised and validated survey tools were used, but also single items.

A significant increase of the proportion of children and adolescents with reduced health-related quality of life compared to the pre-pandemic period was reported from analyses of the study types A and B from the COPSY study for the period of the first wave of the pandemic, both at the federal level [24–26] and for the city and federal state of Hamburg [27]. Children, who belonged to a risk group, formed from being part of families with low education, migration background, or cramped living conditions, were significantly more affected [24–27]. Compared to the pre-pandemic period, the general state of health had not changed [27]. However, declines in the subjectively assessed general activity among 14- to 17-year-olds was reported from the pairfam study (study type B) [28]. Results from the second survey wave of the COPSY study from December 2020 until January 2021, conducted in parallel to the second wave of the pandemic, showed a further decline of the health-related quality of life compared to the first COPSY wave, but with negligible effect sizes [29]. Results from the study ‘Präventionsradar’ (Prevention Radar) of the Institute for Therapy and Health Research (IFT-Nord; Study type C), a long-term study in 13 states with more than 14,000 children and adolescents from 897 classes, showed a decline of life satisfaction among pupils in grades 5 to 10, on average by approximately 21% [30] in the initial phase of the pandemic. Declines of life satisfaction compared to the pre-pandemic period were also reported from the BerO study (study type B) in the spring of 2020 for high school graduates [31] as well as further declines in the course of the pandemic from the spring to the autumn of 2020 [32]. The SPATZ study, a type C birth cohort study, which is representative for the city of Ulm, found declines in the quality of life among children between the ages of 6 and 7 only among girls [33]. Differences in the self-awareness and perception of others with regard to the wellbeing among children and adolescents between the ages of 10 and 17 were reported from a nationwide survey on behalf of the statutory health insurance fund DAK-Gesundheit (study type C) during the first wave of the pandemic. According to the perception of the parents, the wellbeing decreased among 38% of the children and adolescents, and increased among 21% [34]. Using self-report data from the same study, however, only 29% reported a decline of the wellbeing, but 31% reported an increase [34]. As to health-related quality of life, declines are likewise reported from a special COVID-19 study of April 2020 by the Motorik-Modul (MoMo; Study type D) in a descriptive manner [35], both for girls and for boys, for younger children (4- to 10-years old) as well as for older children (11- to 17-years old).

With regard to the quality of life, a paper of the study type E did not find any changes between a pre- and post-lockdown group with children and adolescents under the age of 12 [36] for the first wave of the pandemic. In contrast, a study of the type G that collected data from April to the beginning of May 2020 found an intra-individual decline of the quality of life and a deterioration with regard to the emotions, mood, and the overall satisfaction among children between the ages of 3 and 10 during the first lockdown with small to average effect sizes. However, it found also improvements in leisure time activities and family life [37].

#### (b) Indicators of mental distress

Indicators of mental distress were identified in a total of 19 of the publications included in the review on the basis of 15 data bodies. They could be assigned to four constructs: 1. Perceiving stress (such as COVID-19-associated mental stress, sadness or affects), 2. Perceived stress, 3. Loneliness, and 4. (COVID-19-associated) worries (Annex Table 4). In the respective studies, mostly non-validated survey tools in the form of single items were used for the assessment of the indicators of mental stress, oftentimes without citing a specific reference. The use of standardised and validated tools was reported in only five publications.

Regarding the study types A and B, by more than two thirds of the children and adolescents between the ages of 7 and 17 from the COPSY study was reported that they felt stressed by the COVID-19 pandemic, both for the city and federal state of Hamburg (study type A) and nationwide (study type B) in the initial phase of the pandemic (from May to July 2020) [24–27]. For 7- to 17-year-old children and adolescents from families with low education, migration background and/or with cramped living conditions (<20m^2^ living space/person), a higher stress was reported from May to June 2020 [24, 26]. However, for the period between May and July 2020-the time of the summer plateau 2020-a decline in perceived stress compared to the pre-pandemic period (October 2018 to August 2019) was described from the pairfam study (study type B) for 14- to 17-year-old adolescents [28]. With regard to feelings of loneliness among adolescents between the ages of 13 and 17, an increase compared to the pre-pandemic period was reported from the pairfam study [28, 38]. During the second wave of the pandemic from November 2020 to January 2021, further increases in perceived stress up to a proportion of four fifth of the children and adolescents between the ages of 7 and 17 was reported from studies of the study type B (COPSY study) [39]. An increase in the perception of stress by approximately one third to above 50% was reported by high school graduates in a survey conducted in six federal states (BerO study) [32].

Experiencing a large amount of stress was also reported from papers of the study type C. At the time of the first lockdown (May 2020), 42% of the parents of 10- to 17-year-old children and adolescents indicated in a nationwide representative survey that the Corona crisis stressed their child strongly or very strongly, respectively [34]. In a retrospective survey by the German Network of Academic Medical Research (NUM), conducted from November 2020 to April 2021 in parallel to the second wave of the pandemic, the proportion of children and adolescents between the ages of 11 and 17, who-according to self-reporting-were mentally stressed, was approximately two thirds in a population-based (pre-)school sample. According to information from parents, in contrast, approximately four fifths of the participants felt stressed [23]. In the same sample, decreases of stress among children and adolescents before and during the pandemic were reported in the amount of 11% (self-report) and 4% (parents’-report), respectively. Similar results were specified for a clinical sample from paediatric mental health clinics which was analysed comparatively in the same publication [23]. According to a nationwide representative study of the type C on behalf of the statutory health insurance fund DAK-Gesundheit, almost two thirds of the children and adolescents between the ages of 10 and 17 worried about the impacts of the COVID-19 pandemic, with regard to their own Coronavirus infection, or the infection of someone close to them, during the first wave of the pandemic [34]. Between 2020 and 2021, in the prevention radar of the IFT-Nord (study type C) almost half of the surveyed 14,287 pupils in grades 5 to 10 from a total of 897 classes in 13 federal states reported that they felt stressed often to very often [30]. On the basis of a validated inventory, from a nationwide longitudinal study with a representative initial sample, conducted by the University Medical Centre Hamburg-Eppendorf in cooperation with the German Centre for Addiction Research in Childhood and Adolescence (study type D), an intra-individual increase in the perception of stress among children and adolescents between the ages of 10 and 17 was reported during the first wave of the pandemic until April 2020 compared to the pre-pandemic period (September 2019) [40].

Results relating to the perception of stress as well as relating to social isolation and loneliness were reported from type E studies. Compared to the pre-pandemic period in a study by the Department of Paediatric Psychiatry at the University of Dresden including former patients as well as their families, increases in mental stress in the form of worries, fears, and emotions among children and adolescents with and without mental problems between the ages of 1 and 17 were reported at the beginning of the pandemic (beginning of April to beginning of May 2020). Among children and adolescents without mental problems, reported increases were even stronger [41]. According to a cross-sectional study of the Ludwig-Maximilians-Universität München with non-random convenience sampling (study type E) with data acquisition from the end of April to the beginning of May 2020, mental stress was found among half of the participating 3- to 10-year-old children [37]. In contrast, the study ‘Kind sein in Zeiten von Corona’ (‘Being a child in times of Corona’, study type E), performed between mid-/end of April and mid-/end of May 2020, reported that the majority of the children between the ages of 3 and 15 handled the Corona crisis rather well or well [42]. Particularly in families with high parental educational status, more parents reported that their children handled the pandemic well, compared to families with low education of the parents [42]. Almost one third of the surveyed parents of children and adolescents between the ages of 3 and 15 also reported that their child felt lonely during the pandemic [42]. For children from families with difficult financial situation, loneliness was reported more frequently than for children from families with good financial situation [42]. According to a further analysis of the study type E, conducted in the period between April and June 2020 among children and adolescents between the ages of 5 and 19 with and without mental health problems, a proportion of approximately two thirds felt socially isolated due to the pandemic [43]. In an additional survey as part of the school entry health examinations in the region of Hanover (study type F), an increase of fears as well as an increase in the prevalence of sadness and fits of rage (retrospectively) compared to the pre-pandemic period was reported among school starters from September 2020 to February 2021 – approximately the period of the second wave of the pandemic [44]. With only a few percentage points, however, the increase was comparatively small [44]. In the LIFE Child Study, a longitudinal study of the study type G with non-representative non-random sampling of the initial sample and three survey times conducted in the region of Leipzig, the proportion of children between the ages of 9 and 19, who had no contact with their friends, rose from 3% to 13% (T1) or 14% (T2), respectively (T0: before the pandemic, T1: last week in March 2020, T2: last week in April 2020). The region, however, was affected only little by COVID-19 infections at that time [45]. In that study, approximately 80% of the children missed also personal contacts with their friends [45]. With regard to fears and worries relating to COVID-19, most of the children worried more about the health of their families than of their own. Sixty percent worried at least moderately about the international situation, 20% were afraid of COVID-19 themselves [45]. It was furthermore reported that the proportion of children who believed that the situation would never be the same again after the pandemic, more than doubled within one month (from T1 to T2), from approximately 7% to approximately 16% [45]. In a diary study on the mental effects of homeschooling (study type G) performed with parents of 6- to 19-year-olds from March 27 to April 3, 2020, less positive affect and more negative affect of their children was reported on days when schoolwork had to be done or when the parents were concerned directly with the learning, than on days when this was not the case [46].

#### (c) Indicators of acute symptoms of a mental disorder

In a total of 16 of the included publications, stemming from 11 data bodies, tools and inventories for the screening of general psychopathological symptoms as well as those focussing on specific mental health problems and disorders were used. These could be assigned to five contextual constructs:

1. Psychopathological symptoms in general
2. Psychosomatic problems
3. Eating disorders
4. Depressive symptoms
5. Symptoms of an anxiety disorder.

In these studies, standardised and validated survey tools or inventories, respectively, were used. Only one study [44] did not. In one publication [47], modified items of various subscales of a validated inventory (Strengths and Difficulties Questionnaire; SDQ) were combined to form a new, non-validated total score (‘problem behaviour’). In another publication, the subscale of a validated inventory (State-Trait Depression Scales; STDS) was recoded and was provided with a reverse meaning (from ‘positive mood’ to ‘anhedonia’, i.e. no longer being able to experience joy) [38].

**Figure 2:**
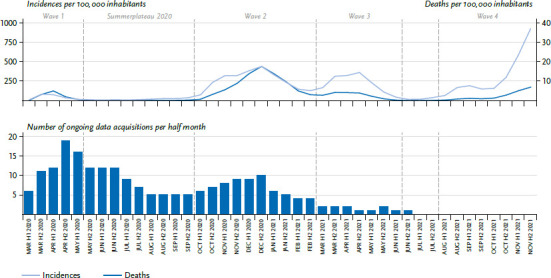
Number of ongoing data acquisitions for each half month of the publications included in the review and development of the COVID-19 pandemic in Germany by incidence and deaths per 100,000 inhabitants Source: Reported SARS-CoV-2 cases to the RKI/own research

Compared to the pre-pandemic period, partially significant increases in the prevalence of general psychopathological symptoms [24–27, 44] as well as of depressive symptoms [24–29, 38, 48] and of anxiety disorder symptoms [24–27] were reported for the initial phase of the pandemic from the studies of type A and B. Almost a doubling of the prevalence of mental health problems (SDQ) for 7- to 17-year-olds from a total of 17.6% in the pre-pandemic period to 30.4% in May/June 2020 was reported nationwide from the first wave of the COPSY study [24–26]. However, no prevalence changes were reported for the city of Hamburg (COPSY Hamburg study) [27]. However, the survey period was later here, from mid-June to mid-July 2020, and was already characterized by a first decline of the COVID-19 incidences (Figure 2) and – associated therewith – relative easing of the strict containment measures after the first wave of the pandemic. For a risk group of children and adolescents from families with low education, migration background and/or with cramped living conditions (<20m^2^ living space/person), more mental health problems were reported in COPSY wave 1 than for children and adolescents, who do not belong to this group. Members of the risk group reported more emotional and conduct problems, hyperactivity and more peer relationship problems, in each case with medium to high effect sizes [26]. In the second survey wave of the COPSY study (study type B) from mid-December 2020 to the end of January 2021, parallel to the second wave of the pandemic (see Figure 2). At 30.6%, the nationwide prevalence of mental health problems remained high [25]. The routine parent survey as part of the school entry health examinations in the region of Hannover (study type A), showed a continuous increase in mental health problems among school starters of the school starter cohorts 2017/2018 to 2020/2021, but at a much lower level (from 5.5% in 2017/2018 to 8.0% in 2020/2021) [44]. For the study types A and B, reports about increases of psychosomatic problems during the first wave of the pandemic, in particular with regard to irritability, bad mood, headaches and abdominal pain, are available from the COPSY study both for the nationwide sample and for the Hamburg sample [24, 27]. Here, children and adolescents from a risk group (i.e., families with low education, migration background and/or cramped living conditions (<20m^2^ living space/person)) were again reported with more psychosomatic problems [24, 26]. In December 2020/January 2021, during the second wave of the pandemic, additional slight increases of psychosomatic problems were reported, but with negligible effect sizes [39]. With regard to depressive symptoms, information is available from the papers of the study types A and B from seven publications [24–28, 38, 48] on the basis of two data bodies (COPSY study and pairfam study). From the pairfam study with an additional COVID-19 survey among 14- to 17-year-old children and adolescents for the period between mid-May 2020 and mid-June 2020, mean value changes for anhedonia and negative mood were reported, which indicate increases in depressive symptoms (compared to mid-October 2018 to mid-August 2019) [38]. With regard to the prevalence of clinically relevant depressive symptoms, increases by 150% (from approximately 10% to approximately 25%) were reported for the same period [28, 48], with significantly higher increases among girls than among boys [48]. In contrast, no increases of depressive symptoms could be determined in the COPSY study from the pre-pandemic period until wave 1, but there was a slight increase (from 11.3% to 15.1%) from the first survey wave from the end of May to mid-June 2020 to the second wave from mid-December 2020 to mid-January of 2021, but with negligible effect sizes. As to the symptoms of a generalised anxiety disorder, however, partially significant increases were reported on the basis of various measuring tools, both nationwide (14.9% to 24.1%) [26] and for the city and federal state of Hamburg (from 15% to 26%) [27]. A further prevalence increase to 30.6% was measured nationwide in the second survey wave of the COPSY study from mid-December 2020 to the end of January 2021 [39].

Studies assigned to the study type C with retrospective surveys partially arrive at the same, partially at different results with regard to general mental health problems as the studies of type A and B. While no increase of mental health problems and only small associations between COVID-19-related stress and mental health problems were reported from an analysis by the Network of Academic Medical Research (NUM) with a (pre-)school sample of 4- to 19-year-old children and adolescents [23], a birth cohort study from Ulm among 6- to 7-year-olds found strong mean value increases, which were interpreted as increase of mental health problems, only among girls [33]. In the study ‘Prevalence Radar’ of the IFT-Nord, in which the emotional problems scale of the SDQ was deployed, increases of emotional problems from a total of 9.2% during the 2018/2019 school year to 13.9% during the 2020/2021 school year were reported descriptively [30]. The increase was stronger among girls, from 14.9% to 22.9%, which corresponds to a relative increase by 53%. Among boys, the frequency increased from 3.8% to 5.5%, thus by 45% [30]. A publication of the study type D between mid-May and the end of July 2020 reported descriptive mean value increases among children between the ages of 5 and 7 for emotional problems, hyperactivity, and conduct problems [49].

Compared to a pre- and post-lockdown group, a study of the study type E with children and adolescents under the age of 12 did not find any differences with regard to mental health problems, eating disorder symptoms, and depressive symptoms as part of a cross-sectional survey. However, a decline with regard to the subscale conduct problems of the SDQ as well as a decline of the number of planned suicides was reported in the survey period from mid-March 2020 to the end of August 2020 (compared to a period from the end of November 2018 to mid-March 2020) [36]. A longitudinal study of the study type G on a non-representative sampling basis found intra-individual declines of ‘problem behaviour’ during the first lockdown (data acquisition from the end of April to the end of May 2020) as well as additional declines during the time of the first easing (mid-July 2020) [47].

#### (d) Indicators relating to the experience of violence in the context of the pandemic

Indicators relating to the experience of violence by children and adolescents in the context of the pandemic could only be found in one of the total of 39 publications included in the review from one data body. The two indicators identified in this study were both assigned to the construct ‘experience of violence’. A single item as well as a list experiment was used for the survey. In the case of a list experiment, participants of a study are randomly assigned to one of two lists. One list (reference group) includes four general questions, the second list (treatment group) includes the same four questions, plus a sensitive item. The participants have to specify in each case, how many of the items apply to them (e.g. three of five). A conclusion can be drawn from the comparison of the average total number between reference and treatment group to the prevalence of the subject matter surveyed with the sensitive item, which is less readily accessible to the direct survey (e.g. experiencing sexual or severe physical violence).

In the study of the type C, performed from April to May 2020 by the Technical University of Munich and the Leibniz Institute for Economic Research in Essen, a prevalence of approximately 6.6% was reported for physical punishment among children and adolescents during the first phase of the pandemic, for children of women with a high-risk profile of almost one fourth (23.3%). If children under the age of 10 lived in the household, the risk for physical punishment increased fivefold [50]. High depression and anxiety values of the respondents and/or of the respective partners increased the risk for physical punishment of the child. A prevalence of approximately 2% was reported for severe physical violence against children during the pandemic [50].

#### (e) Indicators from routine data

Indicators from routine data were reported in five publications from three data sources. They were assigned to three care areas: 1. Outpatient care (two publications from one data source), 2. Inpatient care (one publication from one data source), as well as 3. Child protection (two publications from two data sources). Key figures, such as the number of treatment cases, utilising outpatient medical care due to mental or behavioural disorders, the number of acute or latent cases of child endangerment, as well as the number of the child protection cases reported by schools or day-care centres, were evaluated.

The results from the routine data on the outpatient care of individuals with statutory health insurance utilising care provided by SHI-accredited physicians, are based on the data from 16 of the total of 17 Associations of Statutory Health Insurance Physicians (without Mecklenburg-Western Pomerania) and are provided by the Central Research Institute of Ambulatory Health Care in Germany (Zi) [51, 52]. In the DAK ‘Kinder und Jugendreport 2021’, outpatient and inpatient health care utilisation is presented jointly [17]. They are reported in the outpatient care section here because the number of outpatient treatment cases exceeds the number of inpatient treatment cases. Utilisation data for groups of medical specialists who are primarily tasked with caring for children and adolescents with mental health problems and disorders, is reported below: paediatricians as well as paediatric psychotherapists [vgl. 53].

In April and May 2020, in the early phase of the pandemic, compared to the corresponding months of 2019 or 2018/2019, respectively, billing data for the outpatient and inpatient area show significantly declines in the paediatric, child and adolescent psychiatric as well as a child and adolescent psychotherapeutic treatment cases by up to one third [17, 51]. Catch-up effects in similar magnitudes are reflected in June 2020 with increases compared to the corresponding period. Parallel to the second wave of the pandemic, a decline of the case numbers occurred again in the last quarter of 2020. With regard to utilising paediatric services, there were strong declines again for the first half of 2021 for January, February, April, and May, whereas there were strong increases of the treatment cases for March and June 2021, compared to the pre-pandemic period. This is likewise reported for the number of the paediatric psychiatric treatment cases as well as for the paediatric therapeutic treatment cases, while the level of the latter fluctuated in the remaining months around the respective corresponding pre-pandemic period [17, 51]. Based on the paediatric utilisation, the decline at the preschool and primary school age (up to the age of 9) was lower than among older children and adolescents (between the ages of 10 and 17), here compared to the numbers for 2020 with pooled utilisation data from 2018/2019 [17]. However, a reversed usage pattern was reported for the utilisation of psychiatrists or psychologists, respectively: Here, the reported usage declined during the first lockdown among the children of primary school age, while it increased among older children and adolescents. After the first lockdown and during the second lockdown, increases of the utilisation rates were recorded here among all age groups [17].

Based explicitly on diagnostic data for mental and behavioural disorders (ICD-10: F00-F99), a decline of outpatient doctor’s visits by a total of 11% of statutorily insured children between the ages of 0 and 12 between the second quarter of 2019 and the second quarter of 2020 was determined in an analysis on the basis of the nationwide billing data from the National Association of Statutory Health Insurance Physicians. The decline was smaller for preschoolers than for school-age children [52]. Likewise based on the ICD-10 diagnostic group F00-F99, no differences between 2018, 2019, and 2020 were reported in the DAK Kinder- und Jugendreport 2021 for an age group between 0 and 17 years. Based on the prevalence and incidence of the 10 most frequent disorders, the number of the combined outpatient and inpatient treatment cases in 2020 did not differ from those of the previous year, with respect to the year as a whole [17].

With regard to the inpatient care, an increase of the hospitalisation rate for eating disorders (bulimia and anorexia) among the 5- to 17-year-old children and adolescents by 16.3% compared to the period of the previous year was reported from the data of the statutory health insurance fund DAK-Gesundheit during the first lockdown of 2020 (11.–17. calendar week). While there was still an increase by 3.2% after the first lockdown (18.–44. calendar week), this increase was 26.1% during the second lockdown (45.–52. calendar week). Based on all of 2020, the number of the eating disorder cases treated on an inpatient basis was 9% above the previous year [17]. In the case of depression and anxiety disorders (reported in combination), in contrast, a decline of the hospitalisation rate was determined for the 10- to 17-year-olds during the first lockdown of 2020 (-37%) and a rather slight increase after the first and during the second lockdown compared to the previous year [17]. Based on all of 2020, the hospitalisation rate for depression and anxiety disorders compared to 2019 remains unchanged [17].

The child protection cases reported to child protective services by kindergartens during the first lockdown in April 2020 decreased by one third, the child protection cases reported by schools by more than half [54]. The acute and latent cases of child endangerment reported by child protective services to the Federal Office of Statistics increased significantly from 2019 to 2020. In comparison, the increase was 9% [55]. However, there were already increases by 10% each in 2018 und 2019, so that this development appears to follow a more general trend.

#### (f) Indicators from care-related primary data acquisitions

Results relating to indicators from primary data acquisitions relating to care are taken from seven publications from six data bodies. They can be assigned to three care areas: 1. Outpatient care (two publications from two data bodies), 2. Inpatient care (one publication from one study), and 3. Child protection (four publications from three studies). Publications from the study types B, C, and F are available with regard to the care-related primary data.

For the area of outpatient care, a decline of the utilisation of paediatric care is consistently reported from the Disease Analyser Database (IQVIA; study type B) [18], which is considered to be representative, as well as from the network of medical clinics CrescNET [19] (study type F). For the period between April and December 2020, a decline of the paediatric utilisation of 8% for children and adolescents between the ages of 2 and 17 is reported from the IQVIA database [18], furthermore an almost doubled prevalence for diagnoses of depression as well as for anxiety disorders, with disproportionate increases among girls [18]. According to the data from the CrescNET, the visits to a paediatrician decreased by 65% in April 2020, compared to the same month of the previous year [19].

For the inpatient area, the PSYCHIATRY Barometer (study type C) reported a significant decline of the occupancy rate of the fully and semi-inpatient paediatric mental health facilities by almost 30% for 2020 compared to the pre-pandemic period in a comprehensive survey of all psychiatric and psychosomatic specialist hospitals as well the general hospitals with psychiatric or psychosomatic specialist departments (participation: n=312) [56]. The vast majority (90%) of the paediatric mental health facilities indicated that a decision against an inpatient treatment was rarely or never made due to the COVID-19 pandemic. Of approximately one third of the surveyed facilities, wards were temporarily closed or combined due to the COVID-19 pandemic.

From a survey of all paediatric clinics (participation: 159 out of 365) in Germany in March and April 2020 relating to child protection, a study of the C type reports a decline of child protection cases by 37% compared to the pre-pandemic period. In the inpatient area, the decline was even stronger than in the outpatient area [57]. In a survey of all child protection services in Germany (n=575; response rate 65%) of the study type C, one fifth of child protection services reported declines with regard to risk reports, and one fourth reported declines with regard to individuals taken into care for the period between the first lockdown in 2020 [58, 59]. For the spring of 2021 (compared to the spring of 2020), an increase in performed home visits from long-term outreach care was reported in a survey by the national centre for early intervention of 82 healthcare professionals (study type F), by 60% of the respondents (family midwives and family, health, and paediatric nurses). One fourth of the professionals did not report a change, 17% reported a decline of the home visits [60].

### 3.4 Course of the pandemic and temporal correspondence of the data acquisitions

Figure 2 shows the course of the pandemic based on the incidence and death numbers due to SARS-CoV-2 since the beginning of the pandemic in March 2020 to the end of the inclusion of published data and publications from the systematic search in this review on 19.11.2021. The observational periods of the data acquisitions, on which the publications included in this review are based, are entered below. The observational periods of most of the data bodies included in this review were between March and May 2020, with a peak in the second half of April and the first half of May 2020 – and thus in the period of the first wave of the pandemic with its comparatively low number of infections and deaths. A second, but significantly smaller accumulation of the number of observational periods can be found in the second wave of the pandemic with its significantly higher infection and death rates starting in October 2020 until the end of February 2021. The third wave of the pandemic March 2021 to June 2021 is hardly reflected in the number of ongoing data acquisitions on the basis of the publications included in this review. No information relating to data acquisitions was available for the fourth wave of the pandemic, starting in mid-August of 2021 until the end of the inclusion period of published study results on 19.11.2021.

## 4. Discussion

It was the goal of this rapid review to combine the available evidence relating to the changes of the mental health of children and adolescents in Germany during the COVID-19 pandemic, to classify the available data sources by their informative value for the general population, to assess the scope and the reliability of the representation of mental health aspects in the included studies and data sources, as well as to analyse how the mental health of children and adolescents is represented in the German research and data landscape in the course of the pandemic. To do so, a total of 39 systematically and manually searched publications from primary data acquisitions among children and adolescents themselves or from their parents as well as from routine data and from care-related primary data acquisitions were included for the period from the beginning of the pandemic mid-March 2020 until 19.11.2021. According to the procedure of the preceding rapid review on the mental health of the adult population in Germany during the COVID-19 pandemic [15], the databases of the included publications were systematised and differentiated in data bodies, which were deemed reliable with regard to their informative value for the general population (study types A–D) or into data bodies, which are only reliable to a limited extent (study types E–G). The state of research developed for this review will be combined below, the results will be classified with regard to the reliableness and representativeness of their database, the operationalisation and validity of the outcomes, as well as the respective observational period and- compared to the results from the adult review, where possible, and conclusions will be drawn.

### Number of studies and ratio of the study types

In spite of a longer inclusion period (the inclusion period of the adult review ended on 31.07.2021 [15]) and in spite of expanded inclusion criteria (school-related publications relating to mental health as well as publications relating to child protection were additionally considered in the child review), a smaller number of publications was found for the childhood and adolescence than for adulthood, even though the inclusion of publications was handled comparatively generous (e.g. studies with participants up to the age of 20 were considered, as well as studies, which did not focus primarily on the changes in the mental health of children during the pandemic, but which provided pre- and post-pandemic results, for example, in the sample description). It is unclear if that reflects a lower (research) interest in the mental health of the child and adolescent population during the pandemic or if this is due to practical research aspects, such as higher ethical and data privacy requirements in studies with minors. However, the ratio between publications with a database that is deemed reliable with regard to representativeness of the general population (study type A–D) and studies based on non-representative samples (study type E–G) was similar as for adults (approximately 2/3 to 1/3) [vgl. 15].

### Spectrum and validity of used indicators

A similarly broad spectrum of used indicators and tools for surveying the mental health as among the adults could be found in the primary data publications relating to the mental health of children and adolescents during the pandemic [15]. They could be assigned to the outcome areas ‘positive mental health’, ‘perceived stress’, ‘symptoms of an acute mental disorder’, as well as ‘experience of violence’. None of the included publications reported resilience factors. Indicators relating to perceived stress were reported most frequently, indicators of acute symptoms of a mental disorder were reported second most frequently, and indicators of positive mental health were reported third most frequently. To survey mental stress in the context of the COVID-19 pandemic, mainly single items were used, often without indication of source. The validity and reliability of such information are limited. They should thus be interpreted carefully. In particular, these results were nonetheless discussed prominently in press releases [e.g. 61, 62]. In contrast, mainly standardised and validated tools were used to survey acute psychopathological symptoms as well as the positive mental health. However, using screening instruments for general or specific psychopathology only symptoms of mental disorders can be detected, they are not suitable for making diagnoses of mental disorders. To do so, more complex psycho-diagnostic interviews would be required, which were not used in any of the studies included in this review, possibly to keep the survey economical. Therefore, based on the included publications, no statement about a possible increase of mental disorders can thus be made. Indicators relating to the experience of violence were reported most rarely (only a single study was found on this topic, which met the inclusion criteria).

### Development of the mental health of children and adolescents during the pandemic

In contrast to the results of the adult review [15], a significant impact of the pandemic on the mental health of children and adolescents in Germany was evident from the results of the studies of the types A–D, which were classified as being reliable with regard to their informative value for the general population and with regard to the survey methodology – both in the overall analysis across all outcome areas and across all indicators. Significant increases of the proportion of youths with limited quality of life, declines in the life satisfaction and the general subjective health, increases of different forms of perceptions of stress, and increases of general psychopathological symptoms as well as increases of symptoms of specific mental disorders compared to the pre-pandemic period were reported from these study for the initial phase of the pandemic. This was true both for boys and for girls, as well as for different age groups. Rates between approximately 50% and 80% of children and adolescents who indicated to be mentally stressed by the pandemic were reported from various studies [24–27, 31, 32, 34, 39], based mostly non-validated on single item survey. Even though prevalence of general mental health problems, which were surveyed using standardised and validated tools, were significantly lower with approximately 30%, they had almost doubled compared to the pre-pandemic period [24–26]. With regard to specific psychopathological symptoms, the findings differed: While one study reported increases of depressive symptoms of 150% [28, 48], another study did not find any changes in depressive symptoms, but significant increases of anxiety disorder symptoms [26, 27]. According to the results of a representative study, one in fifteen children suffered physical punishment during the pandemic and two percent severe physical violence [50]. In total, the results suggest that the mental disorders for children and adolescents varied with the course of the pandemic. For example, mostly smaller stress and mental health problems rates were reported from studies, the survey period of which was in the summer plateau of 2020 with the easing of measures [27]. Vice versa, increased perception of stress and higher mental health problems rates were reported when the survey periods corresponded temporally with the waves of the pandemic and the official containment measures associated therewith [39]. Concerning the publications of the study types E–G with non-representative sampling base, the results were less unambiguous. Some of these publications did not report any changes with regard to the quality of life, general psychopathological symptoms, depressive or eating disorder symptoms before and during the lockdown measures [36]. Partially they even reported declines, for instance with respect to planned suicides or ‘problem behaviour’ [36, 47]. A non-representative study from the beginning of the pandemic found that children and adolescents with pandemic-related stress managed mostly well or very well [42]. Other publications of these study types, in contrast, reported a high level of pandemic-related fears and worries, about loneliness and the feeling of social isolation among children, their desire to see their friends again, as well as negative affect due to homeschooling [41, 45, 46].

In spite of individual differing findings, the results from the publications of the study types A–D suggest a significant increase of mental stress among children and adolescents during the pandemic, in particular during the waves of the pandemic, which were accompanied by official non-pharmaceutical containment measures. The child and adolescent population thus turned out to be more vulnerable than the adult population [15]. The findings from the publications of the non-representative study types E–G vary more strongly but should be interpreted carefully due to incalculable potential biases. Some findings suggest that, overall, children and adolescents themselves possibly rated their pandemic-related stress as slightly less serious than did their parents because in some studies, the stress rates surveyed via self-reporting among children were below those, which were determined via the parents [23].

### Vulnerable groups

In general, there were only few results from the included publications relating to specific sociodemographic groups. For children and adolescents from socioeconomically dis-advantaged families, a lower quality of life, more perceived stress, more feelings of loneliness, and more psychopathological symptoms were reported than for children from socioeconomically better-off families-both from publications of the study types A and B, which are more reliable with regard to population-based statements, and from the non-representative study type E. However, the attributable part of the pandemic could not be determined from these study results. It can be assumed that such differences already existed before the pandemic. The results were furthermore of children with low-education parents reported in part for a combined risk group owith, with migration background, and cramped living conditions, which makes it impossible to quantify specific risks. From non-representative studies, there were indications that the pandemic could also have had relief effects for children and adolescents with pre-existing mental disorders: They were found to have fewer fears and concerns than children and adolescents without mental disorders [23, 41].

On the basis of the included publications, possible differential developments in sociodemographic subgroups cannot be traced reliably. Even though there are indications about health inequities in the context of the mental health of children during the pandemic, a systematic identification of risk groups – requirement for a planning of tailored prevention and health promotion measures to target these groups – is still pending.

### Health care

Results from both the routine and the care-related primary data showed declines of the outpatient utilisation of specialists with subsequent catch-up effects during the first and second wave of the pandemic, whereby an increase of outpatient depression and anxiety disorder diagnoses, in particular among girls, was reported [18, 51]. During the second wave of the pandemic, a similar picture emerged for the outpatient utilisation [51].

Results relating to the inpatient care indicate an increase of the treatment of eating disorders with simultaneous decrease of the treatment of depression and anxiety disorders during the first lockdown of 2020 [17]. At the same time, the occupancy rate of the medical clinics declined by one third [56]. A decline of child protection cases in a similar magnitude, stronger even in the inpatient than in the outpatient area, was reported consistently from a survey of all medical clinics in Germany that care for children, as well as from the Federal Office of Statistics [55, 57], while the numbers of the acute and latent cases of child endangerment reported in 2020 increased by 9% [55].

### Research activities and the course of the pandemic

The number of ongoing data acquisitions (for each half month) was highest during the first wave of the pandemic until and including the summer plateau of 2020 with comparatively low COVID-19 incidences and pandemic-associated deaths. With each new wave of the pandemic, the number of ongoing data acquisitions corresponded less with the infection rates. For the fourth wave of the pande mic, information with regard to ongoing data acquisition relating to the mental health of children and adolescents was not available until the end of the inclusion period of the systematic search for the child review on 19.11.2021. This finding largely corresponds to that from the review on the adult population [15]. Even if making allowances for the fact that studies, even on the basis of routine data, is (can be) always published only with a certain temporal latency, it appears as though the novelty of the pandemic initially triggered a keen research interest, which declined significantly in the further course of the pandemic. This is not least supported by the fact that many ad-hoc studies were carried out at the beginning of pandemic and during the first lockdown [34, 37, 47], but also the numerous spontaneous COVID-19-related additional surveys as part of long-term studies [23, 28, 35, 36, 38, 45], the data acquisitions of which mainly took place at the beginning of the pandemic. This, however, is not unproblematic because effects on the mental health can be expected in particular in the case of chronicity of stressors, and the need for knowledge increases in the temporal course of the pandemic in this respect.

### Limitations and strengths

The focus of this rapid review was on the changes in mental health of children and adolescents in the general population during the course of the pandemic. In contrast to the narrative review from 2020 [4], school-related aspects were only considered when a direct link with the mental health of children and adolescents during the pandemic was established in the publications. Even though health and the school situation of children and adolescents are closely linked, this link is not sufficiently represented in research. In particular, due to the repeated school closures in the course of the pandemic this focus would have been desirable with regard to the mental health of children and adolescents. With the exception of children and adolescents with attention deficit/hyperactivity disorders or eating disorders, no subpopulations were searched deliberately for the review, which is a limitation. Specific sociodemographic subpopulations (e.g. families with low parental education or with migration background), however, were considered, given that they were also considered in the included publications. Other groups, such as children and adolescents with a chronic disease or a disability (special health care needs, SHCN) were not addressed. This may have contributed to the poor risk group identification, whereby no information is available from the systematic literature search and the title/abstract screening that proportionately significant sociodemographic subgroups of the children and adolescent population were overlooked.

The age limit for the study inclusion was set to be 20 so as not to eliminate relevant studies with participants, who were already of legal age in some cases. However, statements about the transition into young adulthood or young adulthood itself cannot be made therewith. The physical and mental health of children and adolescents are closely linked. Chronic physical health disorders such as obesity also have a significant mental health component. However, these were not the subject matter of this review. The younger these children are, the more difficult is the systematic distinction between physical and mental health. This applies in particular for the age of early childhood (<3 years). Primarily family-oriented studies have to be used for information about this age, which is important for the later mental development.

When systemising the study types, not all potential sources of biases were considered (e.g. survey mode, response or drop-out rates) [63]. Furthermore, an established risk-of-bias tool was not used because, as in the case of the review on the adult population [15], systematic biases were the explicit object of the study question of this rapid review. Also, effect sizes were not considered, which is reserved for future monothematic meta-analyses. It was, however, the aim of this review, to provide a systematic, wide-ranging overview. Even if - for good reasons - the COVID-19 pandemic can be considered to be the relevant stressor for the mental health of children and adolescents, specific health trends, which already existed before the pandemic, were neither empirically demarcated from time trends during the pandemic nor discussed in most of the publications. The reported time trends can thus not be interpreted as being caused by the pandemic. Indeed, the results of the nationwide KiGGS long-term study rather suggested prevalence declines rather than increases of population-based mental health problems and disorders among children and adolescents in the decade before the pandemic [53]. Moreover, the mental health of children and adolescents is influenced by many further factors, which in part may interact with the pandemic situation so as to increase to reduce risks (e.g. the family situation). The effects of such interactions, however, are not the subject matter of this review. According to our best knowledge, this rapid review provides the first systematic evidence synthesis of pandemic-related population-based results on the the mental health of children and adolescents in Germany, which can be considered a relevant strength on this review. Additional strengths are the systematic, multi-perspective view on the potential biases and the critical evaluation there of and-based on the comparable methodo logical approach-, as well as the comparability with the results of the rapid review for the adult population [15].

### Summary and conclusions

The majority of the studies performed until the second wave of the pandemic showed a relevant deterioration of the well-being and the mental health of children and adolescents. The fact that the mental health of children and adolescents varies with the course of the pandemic shows that the youths reacted sensitively to the partly drastic changes in their environment. Even though the initial perception of acute stress can be considered an adequate reaction to an extraordinary crisis-such as the COVID-19 pandemic- and resulting mental impairments at the symptomatic level are not identical with manifest mental disorders, the risk of developing a mental disorder increases to the extent that stress and overburdening situations last. Particularly in the light of the long-lasting unfavourable effects of mental health problems in childhood and adolescence [53, 64], the prevention of long-term mental and physical health problems and for promoting mental health in youth is advisable.

This rapid review showed that the scientific interest in the sequelae of the pandemic appears to be more limited for children and adolescents than for adults. The look at the number of ongoing data acquisitions since the beginning of the pandemic revealed a lack of studies on the development of the mental health of children in the course of the pandemic from the first wave to 19.11.2021. In fact, the COPSY study is the only repeatedly conducted primary data collection on a nationally representative basis that comprised a comparatively broad spectrum of standardised and validated indicators and held a specific focus on the mental health of children and adolescents of a broad age spectrum. All other studies – including the studies of types A–D with a methodologically sound database – have relevant limitations in various respects, be it with regard to the sampling coverage (e.g. only certain age cohorts or only locally or regionally limited suitability for representative statements), with regard to the used indicators (e.g. use of predominantly non-standardised single items or the use of only few validated indicators, respectively) or with regard to their study focus (not primarily on mental health). A lack of continuous observation of the population-based mental health of children and adolescents in Germany that already existed before became painfully noticeable during the pandemic. So, routinely conducted trend and cohort studies would be desirable, for example, as part of a mental health surveillance with the help of which the mental health of children could be surveyed during the course of the pandemic and beyond. Not least because the consequences of mental stress often reveal only in the course of the further individual development. Such studies could also serve to evaluate the effects of official containment measures as well as of measures health-promoting and prevention as elements of a future proactive crisis and pandemic management.

## Key statement

- This rapid review is the first to present a systematic evidence synthesis on population-based mental health of children and adolescents in Germany during the pandemic.
- Across all outcomes and indicators a significant increase of mental distress and symptoms can be observed during the pandemic.
- In the last decades before the pandemic, the prevalence of mental health problems in children and adolescents declined.
- The amount of mental distress in children and adolescents varied during the course of the pandemic.
- The relatively small number of studies conducted since the beginning of the pandemic suggests a lack of studies monitoring the mental health in youths during the pandemic.

## Appendix

**Annex Table 1:** Applied search strings in databases Source: own table

Database	Search strategy
Weekly updated database with studies on the COVID-19 pandemic (as of 19.11.2021)	**Search string PubMed 1:** (‘COVID-19 Testing’[MeSH Terms] OR ‘COVID-19 Vaccines’[MeSH Terms] OR ‘COVID-19 Serological Testing’ [MeSH Terms] OR ‘COVID-19 Nucleic Acid Testing’[MeSH Terms] OR ‘COVID-19’[MeSH Terms] OR ‘sars cov 2’ [MeSH Terms] OR ‘SARS-CoV-2 variants’[Supplementary Concept] OR ‘spike protein sars cov 2’[Supplementary Concept] OR ‘Severe Acute Respiratory Syndrome Coronavirus 2’[Supplementary Concept] OR ‘COVID-19’ [Supplementary Concept] OR ‘covid 19 drug treatment’[Supplementary Concept] OR ‘covid 19 serotherapy’ [Supplementary Concept] OR ‘Severe Acute Respiratory Syndrome Coronavirus 2’[Title/Abstract] OR ‘ncov*’[Title/Abstract] OR ‘covid*’[Title/Abstract] OR ‘sars cov 2’[Title/Abstract] OR ‘sars cov 2’[Title/Abstract] OR ‘SARS Coronavirus 2’[Title/Abstract] OR ‘Severe Acute Respiratory Syndrome CoV 2’[Title/Abstract] OR ‘Wuhan coronavirus’ [Title/Abstract] OR ‘Wuhan seafood market pneumonia virus’[Title/Abstract] OR ‘SARS2’[Title/Abstract] OR ‘2019-nCoV’[Title/Abstract] OR ‘hcov-19’[Title/Abstract] OR ‘novel 2019 coronavirus’[Title/Abstract] OR ‘2019 novel coronavirus*’[Title/Abstract] OR ‘novel coronavirus 2019*’[Title/Abstract] OR ‘human coronavirus 2019’[Title/Abstract] OR ‘coronavirus disease-19’[Title/Abstract] OR ‘corona virus disease-19’[Title/Abstract] OR ‘coronavirus disease 2019’[Title/Abstract] OR ‘corona virus disease 2019’[Title/Abstract] OR ‘2019 coronavirus disease’[Title/Abstract] OR ‘2019 corona virus disease’[Title/Abstract] OR ‘novel coronavirus disease 2019’ [Title/Abstract] OR ‘new coronavirus*’[Title/Abstract] OR ‘coronavirus outbreak’[Title/Abstract] OR ‘coronavirus epidemic’[Title/Abstract] OR ‘coronavirus pandemic’[Title/Abstract] OR ‘post acute covid 19 syndrome’ [Supplementary Concept] OR ‘post acute covid 19 syndrome’[All Fields] OR ‘long covid’[All Fields]) AND 2019/12/01:2099/12/31[Date-Publication]
	**Search string PubMed 2:** (‘wuhan’[tiab] or china[tiab] or hubei[tiab]) AND (‘Severe Acute Respiratory Syndrome Coronavirus 2’[Supplementary Concept] OR ‘COVID-19’ [Supplementary Concept] OR ‘covid 19 diagnostic testing’[Supplementary Concept] OR ‘covid 19 drug treatment’[Supplementary Concept] OR ‘covid 19 serotherapy’[Supplementary Concept] OR ‘covid 19 vaccine’[Supplementary Concept] OR ‘coronavirus*’[tiab] OR ‘corona virus*’[tiab] OR ncov[tiab] OR covid*[tiab] OR sars*[tiab])
	**Search string Embase 1:** (‘coronavirus disease 2019’/exp OR ‘severe acute respiratory syndrome coronavirus 2’:ti,ab OR ‘severe acute respiratory syndrome coronavirus 2’/exp OR ‘covid 19’/exp OR ncov*:ti,ab OR covid*:ti,ab OR ‘sars cov 2’:ti,ab OR ‘sars-cov-2’:ti,ab OR ‘sars coronavirus 2’:ti,ab OR ‘sars coronavirus 2’/exp OR ‘severe acute respiratory syndrome cov 2’:ti,ab OR ‘wuhan coronavirus’:ti,ab OR ‘wuhan seafood market pneumonia virus’:ti,ab OR sars2:ti,ab OR ‘2019-ncov’:ti,ab OR ‘hcov-19’:ti,ab OR ‘novel 2019 coronavirus’:ti,ab OR ‘2019 novel coronavirus*’:ti,ab OR ‘novel coronavirus 2019’/exp OR ‘2019 novel human coronavirus*’:ti,ab OR ‘human coronavirus 2019’:ti,ab OR ‘coronavirus disease-19’:ti,ab OR ‘corona virus disease-19’:ti,ab OR ‘coronavirus disease 2019’:ti,ab OR ‘corona virus disease 2019’:ti,ab OR ‘2019 coronavirus disease’:ti,ab OR ‘novel coronavirus 2019*’:ti,ab OR ‘novel coronavirus disease 2019’:ti,ab OR ‘novel coronavirus infection 2019’:ti,ab OR ‘2019 corona virus disease’:ti,ab OR ‘new coronavirus*’:ti, ab OR ‘coronavirus outbreak’:ti,ab OR ‘coronavirus epidemic’:ti,ab OR ‘coronavirus pandemic’:ti,ab OR ‘pandemic of coronavirus’:ti,ab OR ‘severe acute respiratory syndrome coronavirus 2 vaccine’/exp OR ‘covid 19 vaccine’/exp OR ‘coronavirus disease 2019’/exp OR ‘sars-cov-2 vaccine’/exp OR ‘anti-sars-cov-2 agent’/exp OR ‘covid-19 testing’/exp OR ‘sars coronavirus 2 test kit’/exp OR ‘pediatric multisystem inflammatory syndrome’/exp OR ‘long covid:ti,ab’ OR ‘long-covid’:ti,ab OR ‘chronic covid disease’:ti,ab) AND (2020:py OR 2021:py OR 2022:py)
	**Search string Embase 2:** (wuhan:ti,ab OR china:ti,ab OR hubei:ti,ab) AND (‘severe acute respiratory syndrome coronavirus 2’:ti,ab OR ‘severe acute respiratory syndrome coronavirus 2’/exp OR ‘severe acute respiratory syndrome coronavirus 2’ OR ‘covid*’:ti,ab OR ‘covid 19’/exp OR ‘covid 19’ OR coronavirus*:ti,ab OR ‘corona virus*’:ti,ab OR ncov:ti,ab OR covid*:ti,ab OR sars*:ti,ab OR ‘sars coronavirus 2’/exp) **+ and additional manual search/analysis of the preprint servers Arxiv, BioRxiv, ChemRxiv, MedRxiv, Preprints.org, ResearchSquare, SSRN and since the use of preVIEW and EuropePMC also of additional preprint servers.**
Endnote Smartgroups	**Filter 1: Search string ‘mental health’:** Smartgroup 1: Mental OR psychologic OR psychosocial OR psychisch OR depression OR depressive symptoms OR depressive symptomology OR Depressionen OR depressive Symptome OR psychiatric Smartgroup 2: psychiatrisch OR anxiety OR Angst OR anxious OR ängstlich OR Angstsymptome OR resilience OR Resilienz OR life satisfaction OR Lebenszufriedenheit Smartgroup 3: quality of life OR Lebensqualität OR wellbeing OR Wohlbefinden OR stress OR PTSD OR PTBS OR post traumatic stress disorder OR Posttraumatische Belastungsstörung Smartgroup 4: trauma OR traumatic OR suicide OR Suizid OR suicidal ideation OR suicidal OR ADHD OR ADHS OR attention deficit hyperactivity disorder OR Aufmerksamkeitsdefizit-/Hyperaktivitätsstörung Smartgroup 5: Aufmerksamkeit OR eating disorder OR disordered eating OR binge eating OR anorexia OR Magersucht OR gestörtes Essverhalten OR anorexia nervosa OR anorectic OR bulimia
	**Filter 2: Search string ‘school’:** Smartgroup 6: School OR Schule OR school closure OR Schulschließung OR home schooling OR home school OR closed school OR geschlossene Schule OR school lockdown OR Heimunterricht Smartgroup 7: preschool OR Vorschule OR kindergarten OR bullying OR Mobbing OR containment measure OR Eindämmungsmaßnahme OR lockdown
	**Filter 3: Search string ‘domestic violence’:** Smartgroup 8: domestic violence OR Häusliche Gewalt OR child abuse and neglect OR abuse OR neglect OR Kindesmissbrauch OR Kindesvernachlässigung OR Missbrauch Smartgroup 9: Kindstötung OR Misshandlung OR Vernachlässigung OR child protective services OR CPS OR Jugendamt OR Jugendämter
	**Filter 4: Search string ‘children and adolescents’:** Smartgroup 10: child OR Kind OR children OR Kinder OR adolescent OR Jugendliche OR adolescence OR Jugend OR teenage OR teenager
	**Filter 5: Search string ‘Germany’:** Smartgroup 11: German OR deutsch OR deutsche OR deutscher OR Germany OR Deutschland
Database of the World Health Organisation (06.12.2021) https://search.bvsalud.org/global-literature-on-novel-coronavirus-2019-ncov/	((tw:(mental*)) OR (tw:(psych*)) OR (tw:(depress*)) OR (tw:(anxi*)) OR (tw:(resilience )) OR (tw:(satisfaction )) OR (tw:(quality of life)) OR (tw:(stress )) OR (tw:(trauma* )) OR (tw:(suicid*)) OR (tw:(ADHD )) OR (tw:(attention deficit hyperactivity disorder)) OR (tw:(eating disorder )) OR (tw:(disordered eating )) OR (tw:(binge eating )) OR (tw:(anorexia )) OR (tw:(anorexia nervosa )) OR (tw:(anorectic )) OR (tw:(bulimia )) OR (tw:(school* )) OR (tw:(school closure )) OR (tw:(home school*)) OR (tw:(school lockdown )) OR (tw:(preschool )) OR (tw:(kindergarten )) OR (tw:(bullying )) OR (tw:(containment measure )) OR (tw:(lockdown )) OR (tw:(domestic violence )) OR (tw:(abuse )) OR (tw:(neglect )) OR (tw:(child protective services )) OR (tw:(CPS )) ) AND ((tw:(child*)) OR (tw:(adolescen*)) OR (tw:(teenage*)) OR (tw:(minor*)) ) AND ( (tw:(German*)) )
Google search	ENGLISH: (Covid OR Corona OR pandem*) AND German* AND (Mental OR disorder OR wellbeing OR distress OR substance OR missuse OR violence OR abuse) AND (child* OR adolescen* OR teenage*) GERMAN: Covid AND Deutsch* AND Psych* AND Kind* OR Jugend* Krankenkasse OR Krankenversicherung OR Routinedaten AND Corona OR Covid AND Psych AND Kind* OR Jugend*

**Annex Table 2:** Classification of study types Source: own table

		Random sample General population	Random sample from ACCESS panel	Adaptation and design weighting	Non-Probability-Sample	Trend Design	Cohort Design	Identical survey of the outcome over several measurement points
Primary data								
**A**	Representative trend study, based on a comprehensive survey or a repetitive, randomly drawn population-based cross-sectional sample or a population-representative quota sampling	X		in part		X		X
**B**	Representative trend study, based on a repetitive sample drawn randomly or by means of a population-representative quota method from an access panel		X	in part		X		X
**C**	One-time comprehensive survey or one-time cross-sectional study, based on a sample drawn randomly from a reference population or an access panel or a population-representative quota sampling	X		in part				
**D**	Representative longitudinal study that allows conclusions to be drawn about intra-individual population-based changes	X		in part				X
**E**	Cross-sectional data based on a self-selected sample (convenience sample)				X	X		
**F**	Repetitive cross-sectional survey on a non-representative sampling basis				X	X		X
**G**	Longitudinal data, based on a self-selected sample (convenience sample)				X	X		X

**Annex Table 3 Publications:** Source: own table Category I: Primary data on the mental health of children and adolescents in Germany in the context of the pandemic

Study type A: Representative trend study, based on a comprehensive survey or a repetitive, randomly drawn, population-based cross-sectional sample or a population-representative quota sampling				
Study/data basis and publications (including observational period etc.)	Data extraction results			
	Outcome		Operationalisation/Measuring instrument	Results of the studies
**COPSY Hamburg study** Online survey: Survey of parents of children between the ages of 11 and 17 (M=13.77; SD=1.97; 48% female) and of adolescents from the age of 17; Random sample from the Central Population Registry in Hamburg; n=1,195 parents and n=1,484 children and adolescents; Information for n=1,037 from the parent survey as well as the self-survey. **Observational period:** 12.06.2020–31.07.2020 [27] Kaman A, Otto C, Adedeji A et al. (2021)	a	Quality of life	KIDSCREEN-10-Index	**Increase** of the proportion of children and adolescents with reduced health-related quality of life (26%) compared to the pre-pandemic period (15%); larger proportion among girls than among boys (χ^2^(1)=22.683; ϕ=0.15) and 14- to 17-year-olds compared to 11- to 13-year-olds (χ^2^(1)=25.277; ϕ=0.16) with low effect sizes. Risk group of children with parents with low level of education or migration background or with mental stress or with cramped living conditions with higher proportion of low quality of life (32% vs. 25% among those not affected, V=0.08) [27].
	a	Life satisfaction	Cantril Ladder	Lower life satisfaction than the national average (11- to 13-year-olds: χ^2^(4)=66.331; V=0.07; 14- to 17-year-olds: χ(4)=296.157; V=0.19), with low effect sizes each [27].
	a	General health	Single item	93% with excellent to good general health, 7% less good to poor. **Unchanged** compared to the pre-pandemic period [27].
	a	Protection factors and resources	Family climate scales (Schneewind)	Within the risk group, children with strong family bond compared to children with weak family bond more rarely with low quality of life (30% vs. 57%, V=0.20) and mental health problems (12% vs. 55%, V=0.25). 5% of the children with strong compared to 58% with weak family bond have emotional problems (V=0.25), 9% compared to 40% have hyperactivity (V=0.21) [27].
	b	COVID-19-specific perception of stress	Single item (scale from 1 not stressful at all to 5 extremely stressful)	Proportion of the children and adolescents with extremely or somewhat stress: 60% [27].
	c	Mental health problems	Strength and Difficulties Questionnaire (SDQ, total score, parents’ report)	**Unchanged** (17% during, 19% before pandemic); Risk group with more mental health problems (34% vs. 14% of those not affected, V=0.22), in particular emotional problems (30% vs. 12%, V=0.19), hyperactivity (22% vs. 7%, V=0.18) and peer relationship problems (25% vs. 17%, V=0.19) [27].
	c		SDQ, subscale emotional problems	**Unchanged** (15% during, 19% before pandemic); more girls than boys affected (χ(1)=12.245; p=0.001; ϕ=0.11) [27].
	c		SDQ, subscale hyperactivity	**Unchanged** (9% during, 13% before pandemic), boys compared to girls (χ^2^(1)=18.133; p <0.001; ϕ=0.13) 11- to 13-year-olds compared to 14-17-year-olds more strongly affected [27].
	c		SDQ, subscale conduct problems	**Unchanged** (10% during, 15% before the pandemic), boys more likely affected [27].
	c		SDQ, subscale peer relationship problems	**Increase** (19% during, 13% before the pandemic) [27].
	c	Depressive symptoms	PHQ-2	17% of the children and adolescents in Hamburg with depressive symptoms, such as dejection, less interest, and hopelessness [27].
	c	Symptoms of a generalised anxiety disorder	Screen for Child Anxiety Related Disorders (SCARED, subscale generalised anxiety)	**Increase**: 26% during, 15% before the pandemic, more girls and more likely 14-17-year-olds [27].
	c	Psychosomatic problems	HBSC symptom checklist	**Increase** compared to the pre-pandemic period headaches: 52% (pre-pandemic 28%; ϕ=0.20), stomach aches: 42% (pre-pandemic 21%; ϕ=0.18), back pain: 43% (pre-pandemic 26%; ϕ=0.15), feeling low: 47% (pre-pandemic 23%; ϕ=0,21), irritability: 74% (pre-pandemic: 40%; ϕ=0.27), nervousness: 28% (pre-pandemic 24%; ϕ=0.04), and sleeping problems: 63% (pre-pandemic 39%; ϕ=0.19) [27].
**Survey as part of the school entry health examinations in the region of Hanover** Survey study (written questionnaires): Survey of parents of school starters as part of the school entry health examinations (SEU) of the school starter cohorts 2017/18 (n=8,973), 2018/19 (n=8,582), 2019/20 (n=9,704), 2020/21 (n=2,178). SEU represent comprehensive surveys of the school starters for each upcoming school year, but the SEU were interrupted in the cohort 2020/21 due to the pandemic, only a portion of the cohort was analysed after resuming, socially disadvantaged families to an increased extent due to a socio-compensatory approach. **Observational period:** 2019/20, 2020/21 [44] Bantel S, Buitkamp M, Wünsch A (2021)	c	Mental health problems	SDQ	**Increase** of mental health problems: 5.5% (2017/18), 6.2% (2018/19), 7.4% (2019/20), 8.0% (2020/21) [44].

Study type B: Representative trend study, based on a repetitive sample, drawn randomly or by means of a population-representative quota method from an access panel				
Study/data basis and publications (including observational period etc.)	Data extraction results			
	Outcome		Operationalisation/Measuring instrument	Results of the studies
**COPSY study** **First wave:** Online survey: Survey of parents of children between 7 and 17 years (M=12.23; SD=3.30; 50% female; n=1,586) as well as additional survey of children and adolescents between 11 and 17 years (M=14.33; SD=1.86; 51.1% female; 1,040); nationwide representative survey (quota sampling); Comparison possible with BELLA data (from 2017) as well as HBSC data (pre-pandemic). **Observational period:** 26.05.2020 –10.06.2020 [24] Ravens-Sieberer U, Kaman A, Otto C et al. (2020) [25] Ravens-Sieberer U, Kaman A, Otto C et al. (2021) [26] Ravens-Sieberer U, Kaman A, Erhart M et al. (2021) [29] Ravens-Sieberer U, Kaman A, Erhart M et al. (2021) **Second wave:** Online survey: Survey of parents of children between 7 and 17 years (M=12.67; SD=3.29; 49.7% female, n=1,923); Follow-up rate: 85.1%; n=337 newly recruited and additional survey of a total of n=1,306 children and adolescents between 11 and 17 years (M=14.56; SD=2.00; 51.9% female); nationwide representative survey (quota sampling); Opportunity to compare with nationwide representative BELLA data (from 2017) as well as HBSC data and first COPSY survey wave. **Observational period:** 17.12.2020–25.01.2021 [29] Ravens-Sieberer U, Kaman A, Erhart M et al. (2021)	a	Health-related quality of life	KIDSCREEN-10-Index	**Decline** in the quality of life: 40.2% report a low quality of life (self-reported) in COPSY wave 1 [24, 25], in COPSY wave 2 47.7% (i.e. further **decline**, but negligible effect ϕ=0.08) [29], pre-pandemic 15.3% (parents’ report), in COPSY wave 1: 41.9% reduced, 54.9% middle, and 3.2% high quality of life) [25, 26]. Children and adolescents from families with low education, migration background and/or cramped space (<20m^2^ living space/person) experienced a lower quality of life in COPSY wave 1 compared to children and adolescents, who do not belong to this group (d-ES=0.67) [24, 26]. Girls were more likely to report a low quality of life than boys (pre-pandemic und in COPSY wave 1) [26]. Younger people were more likely affected by the **decline**: The proportion with low quality of life increased in COPSY wave 1 among the 11-13-year-olds from 7.7% to 41.3%, among the 14-17-year-olds from 17.1% to 39.3% [26]. Intra-individual **decline** between COPSY wave 1 and COPSY wave 2 [29].
	b	COVID-19-specific perception of stress	Single items relating to changes in the relationships with friends, changes in the learning situation, changes in the family relationships, changes in connection with the Corona crisis as a whole (self-developed), scale from 1=not stressful at all to 5=extremely stressful	70.7% of the children and adolescents felt stressed in the COPSY wave 1 [24-26], further **Increase** in wave 2 to 82.6% (difference to wave 1 with small effect (ϕ=0.15) [29]. Children and adolescents from families with low education, migration background and/or cramped space (<20m^2^ living space/person) experienced more stress in COPSY wave 1 compared to children and adolescents, who do not belong to this group (42.5% vs. 26.7%) [24, 26]. 64.4% perceived home schooling to be exhausting in COPSY wave 1 (**unchanged** in COPSY wave 2: 63.9%); 82.8% had less contact with their friends in COPSY wave 1 (**decline** in COPSY wave 2: To 76.1% with negligible effect ϕ=0.07); Limitations in relationships with friends reported in COPSY wave 1 37.9% (**unchanged** in COPSY wave 2: 39.4%); more quarrels in the family (11-17-year-olds) in COPSY wave 1: 26.2% (**unchanged** in COPSY wave 2: 23.8%) [29].
	c	Mental health problems	SDQ total score as well as subscales emotional problems, conduct problems, hyperactivity, peer relationship problems)	**Increase** from pre-pandemic 17.6% to 30.4% in the total score in COPSY wave 1 [24-26], Cohen’s f^2^=0,04; COPSY wave 2: 30.9% [29]. **Increase** in the subscales in COPSY wave 1: Emotional problems: 13.3%, pre-pandemic 10.2% (**unchanged** among girls: 15.3%, pre-pandemic 13.0%; **Increase** among boys: 11.4%, pre-pandemic 7.4%); Conduct problems: 10.0%, pre-pandemic 6.6% (**increase** among girls: 8.3%, pre-pandemic 5.7%; **Increase** among boys: 11,6%, pre-pandemic 7.4%); hyperactivity: 14.6%, pre-pandemic 7.7% (**increase** among girls: 10.8%, pre-pandemic 5.1%; **Increase** among boys: 18.4%, pre-pandemic 10.2%); Peer relationship problems: 11.5%, pre-pandemic 7.5 (**unchanged** among girls: 9.5%, pre-pandemic 7.4%; **Increase** among boys: 13.5%, pre-pandemic 7.6%) [26]. Children and adolescents from families with low education, migration background and/or cramped space (<20m^2^ living space/person) showed more mental health problems in COPSY wave 1 compared to children and adolescents, who do not belong to this group (d-ES=0.83). They showed more emotional problems (d-ES=0.59), more conduct problems (d-ES=0.84), more hyperactivity (d-ES=0.60), and more peer relationship problems (d-ES=0.47) [26]. Intra-individual **increase** of emotional problems and peer relationship problems between COPSY wave 1 and COPSY wave 2 [4]. Intra-individually **unchanged** for total score and conduct problems, intra-individual **decline** of hyperactivity [29].
	c	Depressive symptoms	Centre for Epidemiological Studies Depression Scale for Children (CES-DC)	**Unchanged** in COPSY wave 1 [24-26] as well as COPSY wave 2 [29] compared to before the pandemic. Subscale ‘I felt sad’ **increase** between COPSY wave 1 and wave 2 from 33.7% to 39.8% [29]. Children and adolescents from families with low education, migration background and/or cramped space (<20m^2^ living space/person) displayed more depressive symptoms in COPSY wave 1 compared to children and adolescents, who do not belong to this group (d-ES=0.64) [24, 26]. Intra-individual **increase** between COPSY wave 1 and COPSY wave 2 [29].
	c	Depressive symptoms	Patient Health Questionnaire (PHQ-2)	**Unchanged** in COPSY wave 1 [24-26] compared to before the pandemic. **Increase** between COPSY wave 1 and COPSY wave 2 from 11.3% to 15.1%, but with negligible effect ϕ=0.01 [29].
	c	Symptoms of a generalised anxiety disorder	SCARED, subscale generalised anxiety	**Increase** from pre-pandemic 14.9% to 24.1% (self-reported) in COPSY wave 1 [24-26], but only negligible effect Cohen’s f^2^=0,01; further **increase** in COPSY wave 2 to 30.1%, but with negligible effect ϕ=0.07 [29]. Children and adolescents from families with low education, migration background and/or cramped space (<20m^2^ living space/person) experienced more psychosomatic problems in COPSY wave 1 compared to children and adolescents, who do not belong to this group (d-ES=0.67) [24, 26]. Intra-individual **increase** between COPSY wave 1 and COPSY wave 2 [29].
	c	Psychosomatic problems	HBSC Symptom Checklist (HBSC-SCL)	**Increase** in COPSY wave 1: Irritability 53.2%, pre-pandemic: 39.8%; Sleeping problems: 43.3%, pre-pandemic: 39.2%; Headaches: 40.5%, pre-pandemic: 28.3%; Feeling low: 33.8%, pre-pandemic: 23.0%; Stomach aches: 30.5%, pre-pandemic: 21.3% [24]. Children and adolescents from families with low education, migration background and/or cramped space (<20m^2^ living space/person) experienced more psychosomatic problems in COPSY wave 1 compared to children and adolescents, who do not belong to this group (d-ES=0.67) [24, 26]. Further **increase** between COPSY wave 1 and wave 2 in headaches (to 46.4%, ϕ=0.06), stomach aches (to 3.4%, ϕ=0.06) and feeling low (to 43.4%, ϕ=0.10) [29]. Intra-individual **increase** between COPSY wave 1 and COPSY wave 2 [29].
**German Family Panel (pairfam) and additional COVID-19 survey** Online survey: Nationwide representative cohort with random register sample with four birth cohorts and a total of approximately n=12,000 participants. Approximately 850 participants (57.3% female) of the youngest birth cohort (2001-2003) between 14 and 17 years participated in the additional COVID-19 survey. Data from the 11. wave serves as corresponding pre-pandemic period (T1). **Observational period**: T1: Mid-October 2018 to mid-August 2019 T2: Mid-May 2020 to mid-July 2020 [38] Alt P, Reim J, Walper S (2021) [28] Bujard M, Von den Driesch E, Ruckdeschel K et al. (2021) [48] Naumann E, von den Driesch E, Schumann A et al. (2021)	a	Activity	Single item (scale from 1 to 5)	**Decline** of the subjective activity compared to the pre-pandemic period (T1: M=3.30 vs. T2: M=2.87) [28].
	b	Loneliness	UCLA loneliness scale	**Increase** of loneliness compared to the pre-pandemic period (T1: M=2.10 vs. T2: M=2.27 [38] and T1: M=2.07 vs. T2: M=2.22) [28]).
	b	Stress	Single item (scale from 1 to 5)	**Decline** of perception of stress compared to the pre-pandemic period (T1: M=2.86 vs. T2: M=2.73) [28].
	c	Depressive symptoms	Subscale Positive mood of the German version of the State-Trait Depression Scales (STDS)	**Increase** of anhedonia compared to the pre-pandemic period (T1: M=1.85 vs. T2: M=2.14) [38]. Girls have stronger **increase** of anhedonia [38].
	c		Subscale Negative mood of the German version der State-Trait Depression Scales (STDS)	**Increase** of negative mood compared to the pre-pandemic period (T1: M=1.74 vs. T2: M=1.91) [38]. Girls have stronger **increase** of the negative mood [38].
	c		German version of the State-Trait Depression Scales (STDS) (cut-off >25 for clinically relevant symptoms)	**Increase** of the prevalence of clinically relevant depressive symptoms during the pandemic by approximately 15% (T1: 10.2% vs. T2: 25.2%) [28] and T1: 10.4% vs. T2: 25.3% [48]). Stronger **increase** among girls (T1: 13%, T2: 35%) than among boys (T1: 7% vs. T2: 15%;) [28, 48] . **Increase** among adolescents with migration background (T1: 11% vs. T2: 33%) [48].
**National study ‘Career orientation: Career and study choice’ (BerO study) by the Institute for Employment Research (IAB)** Survey study (mode not mentioned): Survey of the Institute for Employment Research (IAB) of pupils of the graduation class cohorts 2020 and 2021 from Baden-Württemberg, Bavaria, Berlin, Hesse, Lower Saxony, North Rhine-Westphalia, Saxony, Schleswig-Holstein; n=approximately 7,500 during first survey; Spring of 2020: n=1,079; fall of 2020: n=2,849 **Observational period**: 24.03.2020–03.07.2020 and 16.11.2020–21.12.2020 [31] Anger S, Bernhard S, Dietrich H et al. (2021a) [32] Anger S, Bernhard S, Dietrich H et al. (2021b)	a	Life satisfaction	Single item (scale from 0 to10)	**Decline** of the life satisfaction of a graduation class cohort in 2020 compared to the pre-pandemic period (2019: 7.3 vs. 2020: 6.8) [31]. **Decline** of the proportion of graduates with high life satisfaction during the pandemic (spring of 2020: 71% vs. fall of: 61%) [32].
	b	Mental stress	Hopkins Symptom Checklist (HSCL, cutoff >1.8)	**Increase** of the proportion of those who experience high mental stress (32% to 51%) in the course of the pandemic [32].

Study type C: One-time comprehensive survey or one-time cross-sectional study, based on a sample drawn randomly from a reference population or an access panel or a population-representative quota sampling				
Study/data basis and publications (including observational period etc.)	Data extraction results			
	Outcome		Operationalisation/Measuring instrument	Results of the studies
**DAK study ‘Home schooling in times of Corona’** Online survey: Survey of parents and an associated child between 10 and 17 years (n=1.005). Representative population survey on behalf of the DAK-Gesundheit. The determination of the group of parents or guardians to be surveyed was made via a screening as part of the online panel forsa.ominet. **Observational period:** 07.05.2020 –14.05.2020 [34] DAK-Gesundheit (2020)	a	Wellbeing of the child compared to before the school closure	Single item (parents’ report), scale from 1 ‘significantly better’ to 5 ‘significantly worse’ than before the pandemic	**Decline** of the wellbeing (worse or significantly worse) according to parents’ report among 38%, **increase** (better or significantly better) among 21% [34].
			Single item (self-reported), scale from 1 ‘significantly better’ to 5 ‘significantly worse’ than before the pandemic	**Decline** of the wellbeing (worse or significantly worse) according to self-report among 29%, **increase** (better or significantly better) among 31%. On average, younger individuals felt worse instead of better slightly more frequently, it was the opposite among the older individuals [34].
	b	Stress of the child by Corona crisis	Single item (parents’ report), scale from 1 ‘very strongly’ to 4 ‘not at all’	42% of parents reported that the Corona crisis stresses their child very strongly (9%) or strongly (33%), 57% reported that their child is rather less stressed (53%)or not stressed (4%) [34].
	b	Worries about the impacts of the Corona pandemic	Single item (self-reported), scale from 1 ‘often’ to 4 ‘never’	18% worried often, 42% occasionally, 25% rarely, and 12% never about the impacts [34].
		Worries relating to the Corona infection (self or someone close)	Single item (self-reported), scale from 1 ‘often’ to 4 ‘never’	19% worried often, 41% occasionally, 27% rarely, and 12% never about an infection [34].
**SPATZ Health Study** Survey study (written questionnaires): Birth cohort study 2012 with n=362 as comprehensive survey from the maternity clinic in Ulm, which was the only one at that time (response rate 49%). The sample comprises all children of the follow-up examination among 6- and 7-year-olds of the SPATZ study, for which a questionnaire was completed in the first grade. Compared in cross-sectional analysis, children between 6 and 7 years were divided into four groups, which, before the first school closure in Germany (≤ 15 March 2020) were in first grade (groups 1-3), as well as the children, who were in first grade (group 4) during the pandemic (>15 March 2020). **Observational period:** March-May 2020 [33] Kurz D, Braig S, Genuneit J et al. (2021)	a	Quality of life	KINDL-R Total	**Decline** by 5.5 points only for girls (in analysis adjusted by age and school education of the mother) [33].
	c	Mental health problems	SDQ	**Increase** only for girls by 2.0 points during the pandemic (in analysis adjusted by age and school education of the mother) [33].
**Study of the Network of Academic Medical Research (NUM) with participants of the TEMPO study and the B-FAST project** Online survey: Comparison of a medical clinic sample (TEMPO study) and of a population-based (pre-)school sample (B-FAST project); Medical clinic sample: n=280 participants between 4 and 17 years from paediatric psychiatric outpatient clinics from four university medical centres (Aachen, Charité Berlin, Göttingen, and Cologne), who were registered, newly-assessed or re-assessed or who were further treated in the period from 01.12.2020 to 30.03.2021; Pre-school sample: n=1,958 children and adolescents between 4 and 19 years from a total of 18 selected day-care centres and schools at five locations (Düsseldorf, Heidelberg, Homburg, Cologne, and Munich). The selection ‘aimed at representing regions with various population densities and social contexts’ ([23], p. 2). Inclusion period 09.11.2020-18.04.2021 **Observational period:** Fall of 2020 to spring of 2021 [23] Döpfner M, Adam J, Habbel C et al. (2021)	b	COVID-19-related mental stress	Corona stress questionnaire parents (CBB-E; parents’ report)	Medical clinic sample: **Increase** of the total stress among a total of 72% (strong: 23.6%, somewhat: 48.4%) of the children and adolescents according to parents’ report, among 16.1% **unchanged**, among 11.8% **declines** of the stress (somewhat: 10.6%, strongly: 1.2%) [23]. (pre-)school sample: **Increase** of the total stress among a total of 78.2% (strongly: 16.9%, somewhat: 61.3%) of the children and adolescents according to parents’ report, among 17.6% **unchanged**, among 11.8% **decline** of stress (somewhat: 3.9%, strongly: 0.2%) [23].
	b	COVID-19-related mental stress	Corona stress questionnaire children and adolescents (CBB-KJ; Self-report)	Medical clinic sample: **increase** of the total stress among 65.9% (strongly: 19.1%, somewhat: 46.8%) according to self-report of the children and adolescents (22% **unchanged**, among 12% **decline** of the stress slightly: 9.9%, strongly: 2.1%) [23]. (Pre-)school sample: **Increase** of the total stress among a total of 61.4% (strongly: 7.9%, somewhat: 53.5%) of the children and adolescents according to self-report, among 26.4% **unchanged**, among 12.2% **declines** of stress (somewhat: 10.2%, strongly: 2.0%) [23].
	c	Mental health problems	Child Behaviour Check-list (CBCL) (parents’ report about children)	Clinic sample: Small associations between COVID-19-related mental stress and the CBCL total score of mental health problems (r=0.20) [23].
	c	Mental health problems	SDQ-E (parents’ report)	(Pre-)school sample: Mental health problems according to SDQ-E: 11.4%. Small associations between COVID-19-related mental stress and the SDQ-E total score of mental health problems (r=0.33) [23].
	c	Mental health problems	Youth Self Report (YSR) (self-report children and adolescents)	Medical clinic sample: Small associations between COVID-19-related mental stress and the YSR total score of mental health problems (r=0.25) [23].
	c	Mental health problems	SDQ-S (self-report)	(Pre-)school sample: Small associations between COVID-19-related mental stress and the SDQ-E total score of mental health problems (r=0.22) [23].
	c	Screening of mental disorders according to ICD-10/DSM-5	FBB-SCREEN (parents’ report )	Medical clinic sample: Small associations between COVID-19-related mental stress and the FBB-SCREEN score (r=0.13) [23].
	c	Screening of mental disorders according to ICD-10/DSM-5	SBB-SCREEN (self-report)	Medical clinic sample: Small associations between COVID-19-related mental stress and the SBB-SCREEN score (r=0.36) [23].
**Study by the TU Munich and the Leibniz Institute for Economic research in Essen** Online survey: Study with n=3,818 women between 18 and 65 years, in a partnership, on experiences of violence during the pandemic, women with children n=1,474 of it. Quota sampling from pool of 100,000 survey-ready individuals of a private survey institute. Quotas were applied in terms of German state, age, net household income, education, employment status, and household size, in order to ensure the representativeness of the sample. **Observational period:** 22 April to 8 May 2020 [50] Ebert C, Steinert JI (2021)	d	Experience of violence	Physical punishment of children in the family (direct question; Tool not identified in more detail)	Prevalence of physical punishment of children in the last month at 6.58% [50]. High values of depression and anxiety of women double the risk for physical punishment of the children (OR=2.07), high depression and anxiety values among the partners triple it (OR=2.71) [50]. Presence of younger children in the household increases the risk for physical punishment of the child fivefold (OR=5.31) [50]. Adjusted prevalence for the physical punishment of their children among women with a high risk profile at almost one fourth (23.32%) [50].
	d	Experience of violence	List experiment for the survey of, e.g., severe physical violence against children.	Prevalence for severe physical violence against children in the last month at 1.97% [50].
**Prevention radar of the Institute for Therapy and Health Research (IFT-Nord) on behalf of the DAK** Online survey: Pupils from 897 classes (n=14,287). A total of n=4,271 fifth and sixth graders, n=5,259 seventh and eighth graders, and n=4,757 ninth and tenth graders from 13 federal states were surveyed. A comprehensive survey of the respective grades took place. Proportion of the female respondents 49%, average age 13.0 years. Weighted by age, sex, and school type. **Observational period:** Wave 1: 2016/2017; Wave 2: 2017/2018; Wave 3: 2018/2019; Wave 4: 2019/2020; Wave 5: 2020/2021 [30] Hanewinkel R, Hansen J, Neumann C et al. (2021)	a	Life satisfaction	Single item (scale from 0 to 10)	**Decline** of the life satisfaction at 58% compared to before the pandemic [30]. Life satisfaction remained **unchanged** among 24% of the respondents compared to before the pandemic [30]. **Increase** of the life satisfaction at 19% compared to before the pandemic [30]. On average, the life satisfaction of the pupils was reduced by 21% [30].
	b	Perceived stress	Single item (scale not mentioned)	45% of the pupils felt stressed often or very often [30].
	c	Mental health problems	SDQ, subscale emotional problems)	Emotional problems total: 2018/2019: 9.2%; 2019/2020: 10.4%; 2020/2021: 13.9% Emotional problems girls: 2018/2019: 14.9%; 2019/2020: 17.6%; 2020/2021: 22.9% Emotional problems boys: 2018/2019: 3.8%; 2019/2020: 3.4%; 2020/2021: 5.5% [30]

Study type D: Representative longitudinal study that allows conclusions to be drawn about intra-individual population-based changes				
Study/data basis and publications (including observational period etc.)	Data extraction results			
	Outcome		Operationalisation/Measuring instrument	Results of the studies
**Longitudinal study ‘Gaming, Streaming und Social Networking during the Corona pandemic’ by the University Medical Center Hamburg (UKE) in cooperation with the German Centre for Addiction Research in Childhood and Adolescence (DZSKJ)** Survey study (online): Representative nationwide longitudinal study with parents and children between 10 and 17 years (n=824; M=13.06; SD=2.40; 46.6% female); Comparison possible with baseline survey before the pandemic (13.9.2019-27.9.2019). **Observational period:** 20.04.2020–30.04.2020 [40] Paschke K, Arnaud N, Austermann MI et al. (2021)	b	Perceived stress	Perceived Stress Scale (PSS-4) (scale from 0 to 16, cut-off for increased stress ≥ 8)	**Increase** among children and adolescents during the first wave of the pandemic to M=6.93 compared to before the pandemic (M=5.53) [40]. Risk factors for increased perceived stress were financial worries, increased perceived stress of a parent, procrastination, limited access to emotion regulation strategies and staying home during the lockdown.
**TRANS-GEN study** Online survey: Mothers with children between 5 and 7 years (n=73) from a birth cohort started in 2013 by the maternity clinic in Ulm with three waves (t0 to t3) with 158 completed mother-child dyads with complete data sets from all three survey waves. **Observational period:** 18 Mai to 31 Juli 2020 [49] Köhler-Dauner F, Clemens V, Lange S et al. (2021)	c	Mental health problems	SDQ, subscale emotional problems	M=6.17 before the pandemic and M=6.93 during the pandemic, no significance test available [49].
			SDQ, modified subscale conduct problems	M=1.43 before the pandemic and M=3.41 during the pandemic, no significance test available [49].
			SDQ, modified subscale hyperactivity	M=2.63 before the pandemic and M=2.97 during the pandemic, no significance test available [49].
**MoMo study** Online survey: Representative nationwide longitudinal study with children and adolescents between 4 and 17 years (n=1,711). Special study to describe the effects of COVID-19 on the relationship between physical activity, screen time, and health-related quality of life. **Observational period:** Pre-pandemic: August 2018–March 2020 (n=2,843) During the pandemic: 20 April 2020–30 April 2020 (n= 1,711) [35] Wunsch K, Nigg C, Niessner C et al. (2021)	a	Quality of life	KIDSCREEN-10	**Decline** of the health-related quality of life in the analysed age groups and among both sexes (only descriptive, no significance test reported) [35]. Age 4 to 10: Boys: (M_preCOVID-19_=44.89; M_during COVID-19_=40.77) and girls: (M_preCOVID-19_=45.49; M_during COVID-19_=41.27) [35]. Age 11-17: boys: (M_preCOVID-19_=43.15; M_during COVID-19_=40.77) and girls: M_preCOVID-19_=45.49; M_during COVID-19_=40.83) [35].

Study type E: Cross-sectional data, based on a self-selected sample (convenience sample)				
Study/data basis and publications (including observational period etc.)	Data extraction results			
	Outcome		Operationalisation/Measuring instrument	Results of the studies
**Study by the LMU Munich University on child wellbeing and problem behaviour during the pandemic** Online survey (self-selected): Survey of parents of 3- to 10-year-old children; Recruiting of the participants, e.g., via social media, e-mail; n=2,672 (data for 3,389 children because answers were partially for more than one child). **Observational period:** End of April to beginning of May 2020 [37] Christner, N., Essler, S., Hazzam, A., & Paulus, M. (2021)	a	Quality of life	Two self-composed scales ‘emotional wellbeing’ and ‘family wellbeing’ with three items each from the KID-SCREEN-52 questionnaire with modified answer scale (from 1 ‘significantly less’ to 7 ‘significantly more’ with 4 ‘no difference’, to represent changes in the quality of life before and during the COVID-19 pandemic).	**Decline** of the quality of life of the children since the beginning of the pandemic: Deterioration of emptions, mood, and the overall satisfaction since the beginning of the pandemic (ds range 0.35–0.41) [37]. **Increase** or improvement of the leisure time and of family life in 2020 (ds range 0.24–0.54) [37].
	b	Stress	Single item (scale from 1 to 4)	Over 50% of the children and adolescents are rather or significantly stressed, irritated, or lonely in 2020 [37].
**Study: Being a child in times of Corona** Online survey: Survey of parents of children between 3 and 15 years, who were recruited as convenience sample using the snowball method; n=12,628 **Observational period:** 22.04.–21.05.2020 [42] Langmeyer A, Guglhör-Rudan A, Naab T et al. (2020)	b	COVID-19-specific perceived stress	Single item	68% of respondents indicate that their children handle the Corona crises rather well or well [42]. 32% of respondents indicate that the non-pharmaceutical containment measures represented stress for the children [42]. In families with high education, more parents report that their child handles the pandemic rather well or very well ((polytechnicall) university degree: 72%; high-school diploma: 65%), than in families with maximally medium education (55%) [42]. 70% of parents of children with siblings in the same household indicate more frequently that their children handle the situation well or very well, than parents, who live alone with their child (66%) [42].
	b	Loneliness	Single item	27% of parents report that their child felt lonely during the Corona crisis [42]. 27% of respondents indicated that their child felt partially lonely, and 33% rather not lonely [42]. 14% of the parents indicated that their child did not feel lonely during the pandemic [42]. In particular in families in difficult financial situation, the feeling of loneliness among children is reported more frequently (48%) than among families with good financial situation (22%) [42]. Parents perceive loneliness more frequently among only children (33%) than among children with siblings in the household (24%) [42]. Parents reported a lower frequency of feelings of loneliness (30%) when their kindergarteners had contact with their kindergarten teachers, than when there was no contact with the facility (24%) [42].
	c	Mental health problems	SDQ (Parents’ report; Subscales emotional problems and hyperactivity)	23% of children have emotional problems [42]. Among 29% of children, parents report hyperactivity [42]. Emotional problems as well as hyperactivity occurs among children during the pandemic more frequently in families that have great difficulties in coping with their income than in families, in which parents indicated coping well with the income (emotional problems: 44% vs. 18%; hyperactivity: 39% vs. 22%) [42]. With regard to hyperactivity, it was shown that in families, in which the highest degree is the (polytechnical) university degree, hyperactivity occurs less frequently (24%) than in families with maximally medium education (40%) [42].
**ProHEAD project** Online survey: Survey of N=5,408 individuals with completed aassessment from a multi-centre consortium study on the longitudinal analysis of mental health of children and adolescents between 12 and 20 years with a representative school sample in 5 German regions of n=15,000, n=324 participants had their assessment after the first lockdown on 16.03.2020. From n=5,084 participants with assessment before the lockdown, a comparative sample of identical size (n=324) was drwan according to age, sex, and school type (n total=648). While the ProHEAD sample claims representativeness, the study sample is a matched convenience subsample of the collected data. **Observational period:** Before the first lockdown: 26.11.2018-13.03.2020 After the first lockdown 18.03.2020-29.08.2020 [36] Koenig J, Kohls E, Moessner M et al. (2021)	a	Quality of life	KIDSCREEN-10 (self-report)	**Unchanged** in pre- and post-lockdown group [36].
	c	Mental health problems	SDQ total score (self-report)	**Unchanged** in pre- and post-lockdown group. Before the lockdown, higher general psychopathology (SDQ) among individuals with low socioeconomic status, effect lower in the post-lockdown group [36].
	c	Mental health problems	SDQ, subscale emotional problems (self-report)	**Unchanged** in pre- and post-lockdown group. Before the lockdown, higher emotional problems among individuals with low socioeconomic status, effect lower in the post-lockdown group [36].
	c	Mental health problems	SDQ, subscale conduct problems (self-report)	**Decline** after the lockdown (M_pre-lockdown_=2.00; M_post-lockdown_= 1.76, also in age-and sex-adjusted analysis [36].
	c	Mental health problems	SDQ, subscale hyper-activity (self-report)	**Unchanged** in pre- and post-lockdown group [36].
	c	Mental health problems	SDQ, subscale peer relationship problems (self-report)	**Unchanged** in pre- and post-lockdown group. Before the lockdown, more peer problems among individuals with low socio-economic status, effect lower in the post-lockdown group [36].
	c	Mental health problems	SDQ, subscale prosocial behaviour (self-report)	**Unchanged** in pre- and post-lockdown group [36].
	c	Eating disorder symptoms	WCS (self-report)	**Unchanged** in pre- and post-lockdown-Gruppe [36].
	c	Eating disorder symptoms	EDE-Q (self-report)	**Unchanged** in pre- and post-lockdown-Gruppe [36].
	c	Depressive symptoms	PHQ-A (self-report)	**Unchanged** in pre- and post-lockdown group. Before the lockdown, connection more depressive symptoms (PHQ-A) among individuals with low socioeconomic status, effect lower in the post-lockdown group [36].
	c	Suicidal thoughts and behaviour	Three items of Paykel Suicide Scale (PSS; self-report)	**Decline** of planned suicides in the post-lockdown group (6.48%; n=21) compared to the pre-lockdown group (2.16%; n=7), OR=0.32, but with very low case numbers, also in age- and sex-adjusted analysis (OR=0.27) [36].
**Study by the University Dresden on emotions and worries during the COVID-19 pandemic** Online survey: Patient families and former study participants from the Department of Child and Adolescent Psychiatry at the University of Dresden; Utilisation sample with n=456, with n=284 being parents. Parents with and without ‘Mental Health Conditions’ (MHC) were surveyed about themselves and their children between 1 and 17 years (with and without MHC). Retrospective comparison of states from the first wave of the pandemic and the pre-pandemic period (n=284). **Observational period:** 04.04.–06.05.2020 [41] Rothe J, Buse J, Uhlmann A et al. (2021)	b	Fears, emotions, and worries	CoRonavIruSHealth Impact Survey (CRISIS)	**Increase** of most of the worries and fears, among adults as well as among children, with MHC as well as without MHC [41]. Stronger **increases** among groups without MHC (adults as well as children). Higher stress levels among adults with children compared to adults without children, and among adults with MHC compared to adults without MHC [41].
**Multinational study on home schooling with participation of the TU Dortmund and the Phillips University Marburg** Online survey: Anonymous multinational digital survey of parents of children and adolescents between 5 and 19 years; Recruiting via social media, schools, parent networks, and parent self-help groups; Differentiation between children with and without MHC; n_Germany_=1.662 **Observational period:** 28 April to 21 June 2020 [43] Thorell LB, Skoglund C, de la Peña AG et al. (2021)	b	Social isolation	Single item (scale from 1=much less than before to 5=much more than before)	**Increase** of the feeling of social isolation (‘more’ or ‘much more’) among 66.3% of the children without MHC and 67.3% of the children with MHC [43].

Study type F: Repetitive cross-sectional survey on a non-representative sampling basis				
Study/data basis and publications (including observational period etc.)	Data extraction results			
	Outcome		Operationalisation/Measuring instrument	Results of the studies
**Additional survey as part of the school entry health examinations in the region of Hanover** Survey study (written questionnaires, self-selected): Survey of parents of school starters of the school starter cohorts 2017/2018 to 2020/2021 (1: n= n=1,238; 2: n=2,049) on the daily life and child wellbeing during the Corona pandemic. **Observational period:** (1) September 2020-November 2020: n=1,238; (2) November 2020-Februar 2021: n=2,049 [44] Bantel S, Buitkamp M, Wünsch A (2021)	b	Fears	Single item (no scale mentioned)	**Increase** of fears (2020: 25%) during the first lockdown compared to the pre-pandemic period [44].
	b	Sadness	Single item (no scale mentioned)	**Increase** of sadness during the pandemic (2020: 27.0% vs. 2020/21: 32.1%) [44].
	b	Fits of rage	Single item (no scale mentioned)	**Increase** of fits of rage during the pandemic (2020: 21.3% vs. 2020/21: 24.9%) [44].
	c	Problems falling asleep and staying asleep	Single item (no scale mentioned)	**Increase** of problems falling asleep and staying asleep (2020: 12.4% vs. 2020/21: 15.3%) during the pandemic [44].
	c	Psychosomatic symptoms	Single item (no scale mentioned)	**Increase** of the number of children with stomach aches, headaches, sickness, or loss of appetite (2020: 6.5% vs. 2020/21: 8%) during the pandemic [44].

Study type G: Longitudinal data, based on a self-selected sample (convenience sample)				
Study/data basis and publications (including observational period etc.)	Data extraction results			
	Outcome		Operationalisation/Measuring instrument	Results of the studies
**PACO study (psychological adjustment to the COVID-19 pandemic)** Online survey: 21-day diary study with pre and post survey with n=562 participants. Recruiting of parents of children and adolescents between 6 and 19 years via social media platforms, no random sample. Subject matter of the study was effects of home schooling on the health of children, no comparisons of pre-pandemic and pandemic period. **Observational period:** 27 March to 03 April 2020, during the pandemic [46] Schmidt A, Kramer AC, Brose A et al. (2021)	b	Affect	7 items: afraid, angry, sad, worried, happy, cheerful, balanced, and relaxed (scale from 1=not at all to 7=very). Formation of two scales: Positive affect (happy, cheerful, balanced) and negative affect (afraid, angry, sad, worried)	More negative parent-child interactions as well as less positive affect from parents and children, and higher negative affect from the children, but no higher negative affect from the parents on days when the children had to do schoolwork. Also days, on which the parents were integrated more strongly into the learning (i.e. the children worked less independently) with more negative parent-child interactions, less positive affect from the parents and children, and more negative affect among parents and children [46].
**Study by the LMU Munich University on parental stress. Parent-child relationships, and child wellbeing during the pandemic** Online survey: Survey of parents and children between 3 and 10 years (T1: n=2,921; T2: n=890). Participants were recruited via online postings, e-mail invitations to families, who are associated with the institute performing the study, and by word of mouth. **Observational period:** T1: End of April to beginning of May 2020 (completely during the time of the first lockdown) T2: Mid-July 2020 (completely during the time of the first easing) [47] Essler S, Christner N, Paulus M (2021)	a	Quality of life	Two self-composed scales ‘emotional wellbeing’ and ‘family wellbeing’ with three items each from the KIDSCREEN-52 questionnaire with modified answer scale (from 1 ‘significantly less’ to 7 ‘significantly more’ with 4 ‘no difference’, to represent changes in the quality of life before and during the COVID-19 pandemic).	**Decline** of the emotional wellbeing from the pre-pandemic period compared to the period of the lockdown, but **increase** of the emotional wellbeing in the period of the first easing (T1: M=3.40; T2 M=4.29) [47]. **Increase** of the family wellbeing from the pre-pandemic period compared to the period of the lockdown, but **decline** of the family wellbeing in the period of the first easing (T1: M=4.21; T2: M=4.04) [47].
	c	Mental health problems	Self-composed total score ‘problem behaviour’ from three sub-scales of the SDQ with modified items (emotional problems, conduct problems, and hyperactivity)	**Decline** of problem behaviour from the pre-pandemic period compared to the period of the lockdown and further **decline** of problem behaviour during the period of the first easing (T1: M=3.47; T2: M=2.86) [47].
**LIFE Child study** Online survey: Initial sample (n_T0_=608) non-random (recruiting via various facilities, such as outpatient clinics, kindergartens and schools, as well as via media advertising on the radio, TV, Internet, public transport) in Leipzig and surrounding areas (affected only little by COVID-19 at the time of the survey); n_T1_=257, n_T2_=257, participation in T1 and T2: n=187 children and adolescents between 9 and 19 years. **Observational period** T0: before the pandemic T1: Last week of March 2020 T2: Last week of April 2020 [45] Vogel M, Meigen C, Sobek C et al. (2021)	a	Quality of life	Subscale physical wellbeing, subscale mental wellbeing, subscale peers and social support from the KIDSCREEN-27	**Decline** of the physical and mental wellbeing and of the perceived social support, stronger effect among children with medium/low socioeconomic status between T0 and T1; **Unchanged** between T1 and T2 [45].
	b	Social isolation	Single item	**Increase** of the proportion of the children with no contact with peers (personally or online) from 3% before the pandemic to 13% and 14% in March or April 2020 [45]. Approximately 80% of the children missed personal contacts to friends [45].
	b	COVID-19-related fears and worries	Single items (scale from 1 not at all to 5 totally)	Two thirds of the children not or little worried about Corona [45]. Most of the children worried more about the health of their families than about their own; 60% worried at least moderately about the international situation, 20% were afraid of COVID-19 themselves [45]. **Increase** of the proportion of children, who believed that they would never be the same again as before COVID-19 from 7.4% at the beginning of the lockdown at the end of March to 16.2% at the end of April [45].

**Table:** Category II: Routine data and care-related primary data

Routine data				
Study/data basis and publications (including observational period etc.)	Data extraction results			
	Outcome		Indicator	Result
**Outpatient care data from the National Association of Statutory Health Insurance Physicians** Data basis: Data from individuals with statutory health insurance (SHI) utilising care provided by SHI-accredited physicians; Data from 16 of the 17 Association of Statutory Health Insurance Physicians (without Mecklenburg-Western Pomerania) from the billing data from the period 1. quarter 2019 to 1. quarter 2021 as well as early information from the billing data from the 2. quarter 2021. Comparisons possible between 2019, 2020, and the first half of 2021, monthly presentation. **Observational period:** 2019, 2020 to first half of 2021 [51] Mangiapane S, Zhu L, Kretzschmann J et al. (2021)	e	Utilisation of outpatient care by paediatricians	Number of treatment cases	**2020:** **Decline** compared to the corresponding months 2019, strongest in April and May 2020 with -34.9% or -19.3%; **Increase** in June with +26.8% (catch-up effect). New **decline** of the case numbers in October (-7.6%), November (-9.5%), and December (-13.8%). In the remaining months, slight fluctuations compared to the corresponding level in 2019 [51]. **1. half of 2021:** Significant **declines** in January (-32.3%) and February (-25.7%), April (-13.8%), and May (-16.8%), strong **increases** in March (+23.6%) and June (+39.6%). Slight fluctuations compared to the corresponding level 2019 in the remaining months [51]. Changes most pronounced among the paediatricians [51].
	e	Utilisation of outpatient care by paediatric psychiatrists	Number of treatment cases	**2020:** **Decline** compared to the corresponding months 2019, strongest in April and May 2020 with -19.1% or -10.9%; Catch-up effect in June with 23.6%. Slight fluctuations compared to the corresponding level 2019 in the remaining months [51]. **1. half of 2021** **Increases** in March (+14.8%) and June (+27.7%), **Decline** in May (-13.1%). Slight fluctuations compared to the corresponding level 2019 in the remaining months [51].
	e	Utilisation of outpatient care by paediatric psychotherapists	Number of treatment cases	**2020:** **Decline** compared to the corresponding months 2019 strongest in April and May 2020 with -12.6% or -11.8%; **Increase** in June with 29.8% (catch-up effect) [51]. **1. half of 2021** Significant **increases** in March (+22.1%), April (+11.5%), and June (+37.0%) **decline** in May (-13.1%) [51]. Slight fluctuations compared to the corresponding level 2019 in the remaining months [51].
**Data from the Association of Statutory Health Insurance Physicians (KBV)** Anonymised analysis of the nationwide data of the children between 0 and 12 years with KBV statutory health insurance in Germany with at least one doctor’s visit between January 2019 and June 2020. 2019: n=8.29 million; 2020: n=8.5 million. Analysis of the frequency of selected ICD-10-diagnosis groups, including F00-F99 (mental and behavioural disorders). **Observational period:** January 2019-June 2020; Corresponding period: 2. quarter 2019 and 2. quarter 2020 [52] Barschkett M, Koletzko B, Spiess CK (2021)	e	Utilisation of outpatient care due to mental disorders	Number of doctor’s visits from January 2019-June 2020	**Decline** of the outpatient doctor’s visits due to all behavioural or mental disorders (F00-F99) by 11% compared to the pre-pandemic period [52]. **Decline** among preschool-age children was smaller than among school-age children [52].
**DAK Kinder- und Jugendreport 2021** Data basis: Anonymised billing data from n=760,023 children and adolescents up to the age of 17 years, who are ensured with the DAK-Gesundheit. The hospital treatments 2019 and 2020 were compared as well as the hospital treatments between spring and fall/winter lockdown in 2020. **Observational period:** All of 2020; Corresponding period 2019 as well as 2018/2019 [17] Witte, Hasemann, Dankhoff et al. (2021)	Prevalence			
	e	Mental and behavioural disorders in total (ICD-10 F00-F99)	Number of outpatient and inpatient treatment cases (per 1,000)	2018: 270 (girls: 237, boys: 302) 2019: 271 (girls 236, boys: 304) 2020: 269 (girls: 235, boys: 301) No significance test available. [17]
	e	Specific developmental disorders of speech and language (F80)	Number of outpatient and inpatient treatment cases (per 1,000)	**Unchanged.** 2018: 104.6; 2019: 108.2; 2020: 110.2 No significance test available. [17]
	e	Specific developmental disorder of motor function (F82)	Number of outpatient and inpatient treatment cases (per 1,000)	**Unchanged.** 2018: 39.4; 2019: 39.9; 2020: 40.1 No significance test available. [17]
	e	Hyperkinetic disorders (F90)	Number of outpatient and inpatient treatment cases (per 1,000)	**Unchanged.** 2018: 40.4; 2019: 40.9; 2020: 40.1 No significance test available. [17]
	e	Other behavioural and emotional disorders with onset usually occurring in childhood and adolescence (F98)	Number of outpatient and inpatient treatment cases (per 1,000)	**Unchanged.** 2018: 39.9; 2019: 33.2; 2020: 33.9 No significance test available. [17]
	e	Emotional disorders with onset specific to childhood (F93)	Number of outpatient and inpatient treatment cases (per 1,000)	**Unchanged**. 2018: 30.9; 2019: 31.8; 2020: 31.5 No significance test available. [17]
	Prevalence			
	e	Reaction to severe stress, and adjustment disorders (F43)	Number of outpatient and inpatient treatment cases (per 1,000)	**Unchanged.** 2018: 28.5; 2019: 28.7; 2020: 27.2 No significance test available. [17]
	e	Mixed specific developmental disorders (F83)	Number of outpatient and inpatient treatment cases (per 1,000)	**Unchanged.** 2018: 21.6; 2019: 22.6; 2020: 22.9 No significance test available. [17]
	e	Unspecified disorder of psychological development (F89)	Number of outpatient and inpatient treatment cases (per 1,000)	**Unchanged.** 2018: 22.6; 2019: 22.1; 2020: 22.0 No significance test available. [17]
	e	Specific developmental disorder of motor function (F81)	Number of outpatient and inpatient treatment cases (per 1,000)	**Unchanged.** 2018: 21.6; 2019: 21.9; 2020: 20.8 No significance test available. [17]
	e	Conduct disorders (F91)	Number of outpatient and inpatient treatment cases (per 1,000)	**Unchanged.** 2018: 20.1; 2019: 20.1; 2020: 19.1 No significance test available. [17]
	e	Eating disorders (F50)	Number of inpatient treatment cases with diagnosis of an eating disorder (Anorexia nervosa and Bulimia nervosa)	**Increase** among 5- to 17-year-olds in 2020 during the first lockdown (11.-17. CW) by 16.3%, after the first lockdown (18.-44. CW) by 3.2%, in the second lockdown (45-52. CW) 26.1%, in all of 2020 by 8.9% compared to 2019 [17].
	e	Depression (F32/F33)/Anxiety disorders (F40/F41)	Number of inpatient treatment cases with depression or anxiety disorders	**Decline** among 10- to 17-year-olds by 37% during the 1. lockdown 2020 [17]. **Increase** by 5.8% and 7.5% after the first or in the second lockdown [17]. In 2020 **unchanged** compared to 2019 [17].
	Incidence			
	e	Specific developmental disorders of speech and language (F80)	Number of incident cases (per 1,000)	**Unchanged.** 2019: 42.2; 2020: 42.3 No significance test available. [17]
	e	Specific developmental disorder of motor function (F82)	Number of incident cases (per 1,000)	**Unchanged.** 2019: 17.9; 2020: 17.9 No significance test available. [17]
	e	Hyperkinetic disorders (F90)	Number of incident cases (per 1,000)	**Unchanged.** 2019: 12.5; 2020: 11.2 No significance test available. [17]
	Incidence			
	e	Other behavioural and emotional disorders with onset usually occurring in childhood and adolescence (F98)	Number of incident cases (per 1,000)	**Unchanged.** 2019: 18.3; 2020: 18.1 No significance test available. [17]
	e	Emotional disorders with onset specific to childhood (F93)	Number of incident cases (per 1,000)	**Unchanged.** 2019: 16.4; 2020: 15.4 No significance test available. [17]
	e	Reaction to severe stress, and adjustment disorders (F43)	Number of incident cases (per 1,000)	**Unchanged.** 2019: 16.8; 2020: 15.4 No significance test available. [17]
	e	Unspecified disorder of psychological development (F89)	Number of incident cases (per 1,000)	**Unchanged.** 2019: 9.2; 2020: 9.3 No significance test available. [17]
	e	Specific developmental disorder of motor function (F81)	Number of incident cases (per 1,000)	**Unchanged**. 2019: 9.4; 2020: 8.4 No significance test available. [17]
	e	Conduct disorders (F91)	Number of incident cases (per 1,000)	**Unchanged**. 2019: 10.0; 2020: 8.8 No significance test available. [17]
	e	Somatoform disorders (F45)	Number of incident cases (per 1,000)	**Unchanged.** 2019: 14.0; 2020: 12.4 No significance test available. [17]
	e	Utilisation of paediatrician	Number of outpatient treatment cases	**2020 annual comparison:** -8.4% compared to 2018/2019; -8.6% compared to 2019 [17]. **2020 by lockdown phases compared to 2018/2019:** -38.0% during the 1. lockdown, -2.0% after the 1. lockdown, -10.4% during the 2. lockdown; -8.4 total [17]. **2020 by lockdown phases and age groups compared to 2018/2019:** 5-9 years: -25.5% during the 1. lockdown; +23.4% after the 1. lockdown; +7.8% during the 2. lockdown; -9.5% total 10-14 years: -17.6% during the 1. lockdown; +35.5% after 1. lockdown; +23.8% during the 2. lockdown; -5.5% total 15-17 years: -15.0% during the 1. lockdown; +37.5% after the 1. lockdown; +28.9% during the 2. lockdown; -0.6% total [17].
	Incidence			
	e	Utilisation of paediatric psychiatrist and psychologist	Number of outpatient treatment cases	**2020 annual comparison:** +1.9% compared to 2018/2019; -3.1% compared to 2019 [17]. **2020 by lockdown phases compared to 2018/2019:** -20.6% during the 1. lockdown, +2.1% after the 1. lockdown, +4.7% during the 2. lockdown; -1.9 total [17]. **2020 by lockdown phases and age groups compared to 2018/2019:** 5-9 years: -9.1% during the 1. lockdown; +23.8% after the 1. lockdown; +22.4% during the 2. lockdown; 6.0% total [17]. 10-14 years: +7.5% during the 1. lockdown; +37.2% after the 1. lockdown; +42.0% during the 2. lockdown; +0.3% total 15-17 years: +12.1% during the 1. lockdown; +39.5% after the 1. lockdown; +49.3% during the 2. lockdown; 4.9% total [17].
**Federal Office of Statistics** Reports from child protective services about child protection cases and child endangerment among day-care and school-age children and adolescents. **Observational period:** All of 2020 [55] Federal Office of Statistics (2021) [54] Federal Office of Statistics (2021)	e	Development of child endangerment	Acute and latent cases	**Increase** of the acute and latent cases of child endangerment (2020: n=60,551; 2019: n=55,527) [55].
	e	Development of child protection cases	Number of child protection cases reported by schools and day-care centres	**Decline** of the number of the child protection cases reported by schools in April 2020 compared to the previous year (2020: n=674; 2019: n=1,435) as well as reported cases by day-care centres (2020: n=267; 2019: n=408) [54].

Care-related primary data				
Study type C One-time comprehensive survey or one-time cross-sectional study, based on a sample drawn randomly from a reference population or an access panel or a population-representative quota sampling				
Study/data basis and publications (including observational period etc.)	Data extraction results			
	Outcome		Indicator	Result
**PSYCHIATRY barometer** Survey study (mode unknown): Survey of all psychiatric and psychosomatic specialist hospitals as well as general hospitals with psychiatric or psychosomatic specialist departments on behalf of the supporters of the German Hospital Institute (DKI) (this includes: German Hospital Association (DKG), the Association of Hospital Directors in Germany (VKD) and the Association of the Leading Hospital Physicians in Germany (VLK)); n=312 facilities participated. **Observational period:** End of October 2020 to the beginning of January 2021 [56] German Hospital Institute (2021)	f	Occupancy rate paediatric psychiatry	Single items (scales not mentioned)	**Decline** of the occupancy rate of the fully inpatient (2020: 68.5%; 2019: 93.3%) and semi-inpatient (2020: 51.9%; 2019: 95.8%) paediatric psychiatry [56]. With rarely or never, a decision against a treatment due to the COVID-19 pandemic was made in 80% of the paediatric psychiatric facilities [56]. 63% of the facilities indicated never or rarely having reduced the occupancy rate per shared room [56]. 33% of the facilities temporarily closed wards or combined wards due to the Corona pandemic, among 33% of the facilities, there were often closures of semi-inpatient rooms [56].
**Study by the University Medical Center Hamburg (UKE)** Online survey: Comprehensive survey of 343 medical clinics caring for children and of medical child protection outpatient clinics throughout Germany on the development of child protection cases during the COVID-19 lockdown. A total of 81 facilities specified total case numbers for March/April 2019 and March/April 2020. **Observational period:** March/April 2020 [57] Heimann T, Ewert J, Metzner F et al. (2021)	f	Number of child protection cases in March and April in 2019 and 2020.	13 self-developed items (scale not mentioned)	**Decline** of the child protection cases as a whole in March/April 2020 (n=702) compared to April 2019 (n=1,118) (-37%) [57], **decline** by 15% (from 454 to 387 cases) in the outpatient area, by 20% (from 307 to 246 cases) in the inpatient area. No significant differences with regard to age groups and the types of the endangerment [57].
**Project ‘Youth welfare and social changes’ at the German Youth Institute (DJI).** Online survey: Nationwide online comprehensive survey of all 575 youth welfare offices in Germany by the German Youth Institute, n=371 youth welfare offices participated (participation rate 65%). The project has been acquiring and analysing data on the situation and development of child and youth welfare since 1992. **Observational period:** 23.04.2020 –12.05.2020 [58] Mairhofer A, Peucker C, Pluto L et al. (2020) [59] Mairhofer A, Peucker C, Pluto L et al. (2021)	f	Endangerment reports	Number of the endangerment reports	Number of the endangerment reports among most youth welfare offices **unchanged** (55%; **Decline** among 25%, **Increase** among 5%, NI 16%) [58, 59].
	f	Individuals taken into care	Number of individuals taken into care	Number of individuals taken into care among most youth welfare offices **unchanged** (66%; **Decline** among 19%, **Increase** among 2%, NI 14%) [59].

Care-related primary data				
Study type F: Repetitive cross-sectional survey on a non-representative sampling basis				
Study/data basis and publications (including observational period etc.)	Data extraction results			
	Outcome		Indicator	Result
**Outpatient care data from the Disease Analyzer Database (IQVIA)** Data basis: Retrospective cross-sectional study with data from individuals using doctors’ practices, which are part of the panel of the Disease Analyzer Database (IQVIA). The study includes the utilisation of paediatricians by children and adolescents between 2 and 17 years with at least one visit between April 2019 and December 2019 (n=454,741) or between April 2020 and December 2020 (n=417,979) in one of 168 paediatric practices in Germany, participating in the IQVIA panel. This corresponds a coverage of approximately 3.5% for paediatric practices. The practices provide all of the information about consultations and diagnoses of the children and adolescents treated there. **Observational period:** April-December 2019 and April-December 2020 [18] Kostev K, von Vultée C, Weber K et al. (2021)	e	Utilisation of outpatient care provided by paediatricians	Number of patients	**Decline** of the utilisation by a total of 8% [18].
	e	Prevalence of outpatient diagnoses of depression in the period April 2020 to December 2020 compared to April to December 2019	Prevalence of diagnosed depression in the participating paediatric practice	**Increase** of the prevalence of diagnosed depression from 0.23% to 0.47% [18]. The strongest **increases** among girls from 0.28% to 0.72% (+132%) [18].
	e	Prevalence of outpatient diagnoses of anxiety disorders in the period April 2020 to December 2020 compared to April to December 2019	Prevalence of diagnosed anxiety disorders in participating paediatric practice	**Increase** of the prevalence of diagnosed anxiety disorders from 0.31% to 0.59% [18]. **Increases** in particular among girls from 0.35% to 0.72% (+106%) [18].
**CrescNet** Data basis: Nationwide 78 of 151 paediatric practices and paediatric endocrinological practices participating in the CrescNet. **Observational period:** Calendar weeks 0 to 26 in 2019 Calendar weeks 0 to 26 in 2020 [19] Vogel M, Beger C, Gausche R et al. (2021)	f	Number of weekly doctor’s visits in paediatric practices	Doctor’s visits per week	**Decline** of the doctor’s visits by 65% in April 2020 [19].
**Healthcare professional survey II by the National Centre for Early Intervention** Online survey: n=82 Family midwives and family, health, and paediatric nurses from long-term outreach care. **Observational period:** 11.05.2021–26.05.2021 [60] Renner I, van Staa J, Neumann A et al. (2021)	f	Home visits	Number of home visits	**Increase** (‘somewhat or significantly’) among 60% of the professionals in spring of 2021 compared to spring of 2020 [60]. **Unchanged** among 25%, 17% reported **decline** [60].

**Annex Table 4 Indicators:** Source: own table Category I: Primary data on the mental health of children and adolescents in Germany in the context of the pandemic

Outcome type a: Indicators of positive mental health				
Study type	Outcome	Inventory/measuring instrument	Data source/study	Publication
**Construct: Quality of life/wellbeing**				
A	Quality of life	KIDSCREEN-10-Index	COPSY Hamburg study	[27]
B	Quality of life	KIDSCREEN-10-Index	COPSY study	[24-26, 29]
C	Wellbeing	Single item (self and parents’ report)	DAK study ‘Home schooling in times of Corona’	[34]
C	Quality of life	KINDL-R	SPATZ health study	[33]
D	Quality of life	KIDSCREEN-10-Index	MoMo study	[35]
E	Quality of life	Self-composed scales ‘Emotional Wellbeing’ and ‘Family Wellbeing’, from three items each from the KIDSCREEN-52	Study by the LMU Munich on child wellbeing and problem behaviour during the pandemic	[37]
E	Quality of life	KIDSCREEN-10	PROHead project	[36]
G	Quality of life	Self-composed scales ‘Emotional Wellbeing’ and ‘Family Wellbeing’, from three items each from the KIDSCREEN-52	Study by the LMU Munich on parental stress, parent-child relationships, and child wellbeing during the pandemic	[47]
G	Quality of life	Subscale ‘Physical Wellbeing’, subscale ‘Emotional Wellbeing’, subscale ‘Peers’, and ‘Social Support’ from the KIDSCREEN-27	LIFE Child study	[45]
**Construct: Life satisfaction**				
A	Life satisfaction	Cantril Ladder	COPSY Hamburg study	[27]
B	Life satisfaction	Single item	BerO study	[31, 32]
C	Life satisfaction	Single item	Prevention radar of the Institute for Therapy and Health Research (IFT-Nord) on behalf of the DAK	[30]
**Construct: General health**				
A	General health	Single item	COPSY Hamburg study	[27]
B	Activity	Single item	German Family Panel pairfam	[28]
**3 Constructs**			**12 Data sources/studies**	**16 References**

Outcome type b: Indicators of mental stress				
Study type	Indicator	Inventory	Data source/study	Publication
**Construct: Perceived stress**				
A	COVID-19-specific perception of stress	Single item	COPSY Hamburg study	[27]
B	COVID-19-specific perception of stress	Single items relating to changes in the relationships with friends, changes in the learning situation, changes in the family relationships, changes in connection with the Corona crisis as a whole	COPSY study	[24–26, 39]
B	Mental stress	Hopkins Symptom Checklist	BerO study	[32]
C	Stress by Corona crisis	Single item	DAK study ‘Home schooling in times of Corona’	[34]
C	COVID-19-related mental stress on children	Corona stress questionnaire according to parents’ report(CBB-E)	Online survey Network of Academic Medical Research (NUM)	[23]
C	COVID-19-related mental stress	Corona stress questionnaire according to self-report (CBB-KJ)	Online survey by the Network of Academic Medical Research (NUM)	[23]
E	Mental stress (worries, sadness, anxiety, restlessness, concentration, irritability, loneliness, negative feelings)	CoRonavIruS Health Impact Survey (CRISIS)	Study by the University Dresden on emotions and worries during the COVID-19 pandemic	[41]
E	Stress	Single item	Study by the LMU Munich on child wellbeing and problem behaviour during the pandemic	[37]
E	COVID-19-specific perception of stress	Single item	Study ‘Being a child in times of Corona’	[42]
E	Social isolation	Single item	Multinational study with participation of the TU Dortmund and the Phillips-University Marburg	[43]
F	Sadness	Single item	Additional survey as part of the school entry health examinations in Hanover	[44]
F	Fits of rage	Single item	Additional survey as part of the school entry health examinations in Hanover	[44]
F	Fears	Single item	Additional survey as part of the school entry health examinations in Hanover	[44]
G	Social isolation	Single item	LIFE Child study	[45]
G	COVID-19-related fears and worries	Single item	LIFE Child study	[45]
G	Affect	Scales on positive and negative affect on the basis of four or three items, respectively	PACO study	[46]
**Construct: Perceived stress**				
B	Perceived stress	Single item	German Family Panel pairfam	[28]
C	Perceived stress	Single item	Prevention radar of the Institute for Therapy and Health Research (IFT-Nord) on behalf of the DAK	[30]
D	Perceived stress	Perceived Stress Scale (PSS-4)	Longitudinal study ‘Gaming, Streaming, and Social Networking during the Corona pandemic’	[40]
**Construct: Loneliness**				
B	Loneliness	UCLA Loneliness Scale	German Family Panel (pairfam) and additional COVID-19 survey	[28, 38]
E	Loneliness	Single item	Study ‘Being a child in times of Corona’	[42]
**Construct: Worries**				
C	Corona-related worries	Single item with regard to effects Single item with regard to infection	DAK study ‘Home schooling in times of Corona’	[34]
**4 Constructs**			**15 Data sources/studies**	**19 References**

Outcome type c: Indicators of acute symptoms of a mental disorder				
Study type	Indicator	Inventory	Date source/study	Publication
**Construct: Psychopathological symptoms in general**				
A	Mental health problems	SDQ (all problem scales, parents’-report)	COPSY Hamburg study	[27]
A	Mental health problems	SDQ	Additional survey as part of the school entry health examinations in Hannover	[44]
B	Mental health problems	SDQ (all problem scales, parents’-report)	COPSY study	[24–26, 39]
C	Mental health problems	SDQ (all problem scales, parents’ report and children self-report)	Study by the Network of Academic Medical Research (NUM)	[23]
C	Mental health problems	CBCL	Study by the Network of Academic Medical Research (NUM)	[23]
C	Mental health problems	YSR	Study by the Network of Academic Medical Research (NUM)	[23]
C	Screening of mental disorders according to ICD-10/DSM-5	FBB-SCREEN (parents’-report)	Study by the Network of Academic Medical Research (NUM)	[23]
C	Screening of mental disorders according to ICD-10/DSM-5	SBB-SCREEN (self-report)	Study by the Network of Academic Medical Research (NUM)	[23]
C	Mental health problems	SDQ (total score, parents’ report)	SPATZ Health study	[33]
C	Mental health problems	SDQ (subscales)	Prevention radar of the Institute for Therapy and Health Research (IFT-Nord) on behalf of the DAK	[30]
D	Mental health problems	SDQ (subscales emotional problems, conduct problems, hyperactivity)	TRANS-GEN study	[49]
E	Mental health problems	SDQ (subscales emotional problems and hyperactivity, parents’ report)	Study ‘Being a child in times of Corona’	[42]
E	Mental health problems	SDQ (all scales, self-report)	ProHEAD project	[36]
G	Mental health problems	Self-composed total score ‘Problem behaviour’ from three problem scales of the SDQ (emotional problems, conduct problems, and hyperactivity) with modified items	Study by the LMU Munich on parental stress, parent-child relationships, and child wellbeing during the pandemic	[47]
**Construct: Eating disorder symptoms**				
E	Eating disorder symptoms	WCS (self-reported)	ProHEAD project	[36]
E	Eating disorder symptoms	EDE-Q (self-reported)	ProHEAD project	[36]
**Construct: Psychosomatic problems**				
A	Psychosomatic problems	HBSC Symptom Checklist	COPSY Hamburg study	[27]
B	Psychosomatic problems	HBSC Symptom Checklist	COPSY study	[24, 39]
F	Psychosomatic problems	Single item	Additional survey as part of the school entry health examinations in Hannover	[44]
F	Problems falling asleep and staying asleep	Single item	Additional survey as part of the school entry health examinations in Hannover	[44]
**Construct: Depressive symptoms**				
A	Depressive symptoms	PHQ-2	COPSY Hamburg study	[27]
B	Anhedonia	German version of the State-Trait Depression Scales (STDS) (Trait-subscale, recoding of the positive mood relating to anhedonia)	German Family Panel pairfam und COVID-19-additional survey	[38]
B	Depressive symptoms	German version of the State-Trait Depression Scales (STDS) (Cut-off >25 for clinically relevant symptoms)	German Family Panel pairfam und COVID-19-additional survey	[28, 65]
B	Depressive symptoms	Patient Health Questionnaire (PHQ-2)	COPSY study	[24–26, 39]
B	Depressive symptoms	Centre for Epidemiological Studies Depression Scale for Children (CES-DC)	COPSY study	[24–26, 39]
E	Depressive symptoms	PHQ-A	ProHEAD project	[36]
E	Suicidal thoughts and behaviour	Three items of Paykel Suicide Scale (PSS)	ProHEAD project	[36]
**Construct: Symptoms of an anxiety disorder**				
A	Symptoms of a generalised anxiety disorder	Screen for Child Anxiety Related Disorders (SCARED, subscale generalised anxiety)	COPSY Hamburg study	[27]
B	Symptoms of a generalised anxiety disorder	SCARED (subscale generalised anxiety)	COPSY study	[24-26, 39]
**5 Constructs**			**11 Data sources/studies**	**16 References**

Outcome type d: Indicators relating to the experience of violence				
Study type	Indicator	Inventory	Date source/study	Publication
**Construct: Experience of violence**				
C	Physical punishment	Direct question about physical punishment of children in the family.	Study by the TU Munich and the Leibniz-Institute for Economic Research in Essen	[50]
C	Severe physical violence against children	List experiment	Study by the TU Munich and the Leibniz-Institute for Economic Research in Essen	[50]
**1 Constructs**			**1 Data source/study**	**1 Reference**

**Table:** Category II: Routine data and care-related primary data

Routine data (type e)			
Indicator	Operationalisation	Data body or owner	Publication
**Care area: Outpatient care**			
Utilisation of outpatient care by paediatricians	Number of treatment cases	Outpatient care data from the National Association of Statutory Health Insurance Physicians	[51]
Utilisation of outpatient care for paediatric psychiatrists	Number of treatment cases	Outpatient care data from the National Association of Statutory Health Insurance Physicians	[51]
Utilisation of outpatient care for paediatric psychiatrists	Number of treatment cases	Outpatient care data from the National Association of Statutory Health Insurance Physicians	[51]
Utilisation of outpatient care due to mental disorders (ICD-10: F00-F99)	Number of doctor’s visits due to mental or behavioural disorders	Outpatient care data from the National Association of Statutory Health Insurance Physicians	[52]
Utilisation of outpatient care by psychiatrist and psychologist	Number of treatment cases	DAK Kinder- und Jugendreport 2021	[17]
Utilisation of outpatient care by paediatricians	Number of treatment cases	DAK Kinder- und Jugendreport 2021	[17]
**Care area: Inpatient care**			
Eating disorders	Inpatient treatment cases with diagnosis of Bulimia nervosa or Anorexia nervosa	DAK Kinder- und Jugendreport 2021	[17]
Depression and anxiety disorders	Inpatient treatment cases with diagnosis of depression or anxiety disorders	DAK Kinder- und Jugendreport 2021	[17]
**Outpatient and inpatient care (pooled)**			
Mental and behavioural disorders in total (ICD-10: F00-F99)	Number of outpatient and inpatient treatment cases (per 1,000)	DAK Kinder- und Jugendreport 2021	[17]
Specific developmental disorders of speech and language (F80)	Number of outpatient and inpatient treatment cases (per 1,000)	DAK Kinder- und Jugendreport 2021	[17]
	Number of incident cases (per 1,000)	DAK Kinder- und Jugendreport 2021	[17]
Specific developmental disorder of motor function (F82)	Number of outpatient and inpatient treatment cases (per 1,000)	DAK Kinder- und Jugendreport 2021	[17]
	Number of incident cases (per 1,000)	DAK Kinder- und Jugendreport 2021	[17]
Hyperkinetic disorders (F90)	Number of outpatient and inpatient treatment cases (per 1,000)	DAK Kinder- und Jugendreport 2021	[17]
	Number of incident cases (per 1,000)	DAK Kinder- und Jugendreport 2021	[17]
Other behavioural and emotional disorders with onset usually occurring in childhood and adolescence (F98)	Number of outpatient and inpatient treatment cases (per 1,000)	DAK Kinder- und Jugendreport 2021	[17]
	Number of incident cases (per 1,000)	DAK Kinder- und Jugendreport 2021	[17]
Emotional disorders with onset specific to childhood (F93)	Number of outpatient and inpatient treatment cases (per 1,000)	DAK Kinder- und Jugendreport 2021	[17]
	Number of incident cases (per 1,000)	DAK Kinder- und Jugendreport 2021	[17]
Reaction to severe stress, and adjustment disorders (F43)	Number of outpatient and inpatient treatment cases (per 1,000)	DAK Kinder- und Jugendreport 2021	[17]
	Number of incident cases (per 1,000)	DAK Kinder- und Jugendreport 2021	[17]
Mixed specific developmental disorders (F83)	Number of outpatient and inpatient treatment cases (per 1,000)	DAK Kinder- und Jugendreport 2021	[17]
Unspecified disorder of psychological development (F89)	Number of outpatient and inpatient treatment cases (per 1,000)	DAK Kinder- und Jugendreport 2021	[17]
	Number of incident cases (per 1,000)	DAK Kinder- und Jugendreport 2021	[17]
Specific developmental disorder of motor function (F81)	Number of outpatient and inpatient treatment cases (per 1,000)	DAK Kinder- und Jugendreport 2021	[17]
	Number of incident cases (per 1,000)	DAK Kinder- und Jugendreport 2021	[17]
Conduct disorder (F91)	Number of outpatient and inpatient treatment cases (per 1,000)	DAK Kinder- und Jugendreport 2021	[17]
	Number of incident cases (per 1,000)	DAK Kinder- und Jugendreport 2021	[17]
Somatoform disorders (F45)	Number of incident cases (per 1,000)	DAK Kinder- und Jugendreport 2021	[17]
**Care area: Child protection**			
Child endangerment	Number of acute and latent cases of child endangerment	Federal Office of Statistics	[55]
Child protection cases	Number of child protection cases reported by schools and day-care centres	Federal Office of Statistics	[54]
**3 Care area**		**3 Data sources**	**5 References**

Care-related primary data (type f)				
Study type	Outcome	Indicator/Operationalisation	Data source/study	Publication
**Care area: Outpatient care**				
B	Utilisation of outpatient paediatric care	Number of doctor’s visits	Outpatient care data from the Disease Analyzer Database (IQVIA)	[18]
B	Depression	Number of diagnoses of depression in paediatric practices	Outpatient care data from the Disease Analyzer Database (IQVIA)	[18]
B	Anxiety disorders	Number of anxiety disorder diagnoses in paediatric practices	Outpatient care data from the Disease Analyzer Database (IQVIA)	[18]
F	Utilisation of outpatient paediatric care	Number of doctor’s visits	CrescNET	[19]
**Care area: Inpatient care**				
C	Occupancy rate paediatric psychiatry	Single items	PSYCHIATRY barometer	[56]
**Care area: Child protection**				
C	Child protection cases	Number of child protection cases, single item	Study by the University Hospital Hamburg Eppendorf (UKE)	[57]
C	Endangerment reports	Number of endangerment reports, single item	Comprehensive survey of youth welfare offices	[58, 59]
C	Taking into care	Number of individuals taken into care, single item	Comprehensive survey of youth welfare offices	[58, 59]
F	Home visits	Number of home visits, single item	Survey of healthcare professional of the National Centre for Early Intervention	[60]
**3 Care areas**			**6 Data sources/studies**	**7 References**
